# Metabolic Features of *Protochlamydia amoebophila* Elementary Bodies – A Link between Activity and Infectivity in *Chlamydiae*

**DOI:** 10.1371/journal.ppat.1003553

**Published:** 2013-08-08

**Authors:** Barbara S. Sixt, Alexander Siegl, Constanze Müller, Margarete Watzka, Anna Wultsch, Dimitrios Tziotis, Jacqueline Montanaro, Andreas Richter, Philippe Schmitt-Kopplin, Matthias Horn

**Affiliations:** 1 Division of Microbial Ecology, Department of Microbiology and Ecosystem Science, University of Vienna, Vienna, Austria; 2 Research Unit Analytical BioGeoChemistry, Helmholtz Zentrum München, Neuherberg, Germany; 3 Division of Terrestrial Ecosystem Research, Department of Microbiology and Ecosystem Science, University of Vienna, Vienna, Austria; University of California Irvine, United States of America

## Abstract

The *Chlamydiae* are a highly successful group of obligate intracellular bacteria, whose members are remarkably diverse, ranging from major pathogens of humans and animals to symbionts of ubiquitous protozoa. While their infective developmental stage, the elementary body (EB), has long been accepted to be completely metabolically inert, it has recently been shown to sustain some activities, including uptake of amino acids and protein biosynthesis. In the current study, we performed an in-depth characterization of the metabolic capabilities of EBs of the amoeba symbiont *Protochlamydia amoebophila*. A combined metabolomics approach, including fluorescence microscopy-based assays, isotope-ratio mass spectrometry (IRMS), ion cyclotron resonance Fourier transform mass spectrometry (ICR/FT-MS), and ultra-performance liquid chromatography mass spectrometry (UPLC-MS) was conducted, with a particular focus on the central carbon metabolism. In addition, the effect of nutrient deprivation on chlamydial infectivity was analyzed. Our investigations revealed that host-free *P. amoebophila* EBs maintain respiratory activity and metabolize D-glucose, including substrate uptake as well as host-free synthesis of labeled metabolites and release of labeled CO_2_ from ^13^C-labeled D-glucose. The pentose phosphate pathway was identified as major route of D-glucose catabolism and host-independent activity of the tricarboxylic acid (TCA) cycle was observed. Our data strongly suggest anabolic reactions in *P. amoebophila* EBs and demonstrate that under the applied conditions D-glucose availability is essential to sustain metabolic activity. Replacement of this substrate by L-glucose, a non-metabolizable sugar, led to a rapid decline in the number of infectious particles. Likewise, infectivity of *Chlamydia trachomatis*, a major human pathogen, also declined more rapidly in the absence of nutrients. Collectively, these findings demonstrate that D-glucose is utilized by *P. amoebophila* EBs and provide evidence that metabolic activity in the extracellular stage of chlamydiae is of major biological relevance as it is a critical factor affecting maintenance of infectivity.

## Author Summary

The *Chlamydiae* are a group of bacteria that strictly rely on eukaryotic host cells as a niche for intracellular growth. This group includes major pathogens of humans and animals as well as symbionts of protists. Unlike most other bacteria, chlamydiae alternate between two distinct developmental stages. Here we provide novel insights into the infective stage, the elementary body (EB), which has been described almost a century ago and is commonly referred to as an inert spore-like particle. Our analyses of EBs of the amoeba symbiont *Protochlamydia amoebophila* provide a detailed overview of their metabolism outside of, and independent from, their natural host cells. We demonstrated that these EBs are capable of respiration and are active in the major routes of central carbon metabolism, including glucose import, biosynthetic reactions, and catabolism for energy generation. Glucose starvation resulted in a rapid decline of metabolic activity in *P. amoebophila* EBs and a concomitant decrease in their potential to infect new host cells. The human pathogen *Chlamydia trachomatis* was also dependent on nutrient availability for extracellular survival. The extent of metabolic activity in chlamydial EBs and its consequences for infectivity challenge long-standing textbook knowledge and demonstrate that the infective stage is far more dependent on its environment than previously recognized.

## Introduction

The *Chlamydiaceae* are a group of obligate intracellular bacteria that have been well-known for more than a century and include some of the most successful bacterial pathogens. Two species in particular are considered to represent a major threat to human health, *Chlamydia trachomatis*, a well-established agent of trachoma and sexually transmitted disease [1], [2], and *Chlamydia pneumoniae*, which causes pneumonia and has also been associated with numerous chronic diseases [3]. More recently, the discovery of the so-called “environmental chlamydiae” has radically changed our perception of chlamydial diversity and distribution in nature. These newly discovered species, which are related to the *Chlamydiaceae* yet represent separate families within the phylum *Chlamydiae*, infect host species as diverse as vertebrates, invertebrates, and even protozoa [4], [5]. Their impact on human health is not yet well understood, though there is evidence that some species might represent emerging pathogens [4], [6]. Among the most well-studied representatives of the environmental chlamydiae are members of the family *Parachlamydiaceae*, in particular *Protochlamydia amoebophila* [7] and *Parachlamydia acanthamoebae* [8]. Although primarily considered to be symbionts of amoebae [4], [9], there are indications that they may be associated with human diseases, and in particular respiratory tract infections [10], [11].

Although we are just beginning to understand aspects of host range, host-symbiont interactions, and potential pathogenicity of these newly discovered species, it is well-established that all characterized environmental chlamydiae share distinctive key aspects of chlamydial biology with the *Chlamydiaceae* [4]. These include a strict dependence on a eukaryotic host cell as a replicative niche as well as a biphasic developmental cycle, both of which have major implications on the metabolic traits of this group of bacteria.

Investigations of the genomic repertoire of several members of the *Chlamydiae* have revealed that they harbor highly reduced metabolic capacities, presumably as a consequence of their adaptation to intracellular life [12]–[18]. Although environmental chlamydiae, including *P. amoebophila*, have a significantly larger genome size than the *Chlamydiaceae*, their metabolic potential is only slightly increased, and they are thus also strictly dependent on eukaryotic host cells [14], [16]–[19]. Despite being auxotrophic for most amino acids, cofactors, and nucleotides and being able to take up host-derived ATP, chlamydiae have at least partially maintained metabolic pathways devoted to carbon metabolism and energy generation [19]–[21]. However, little is known about the role of these metabolic features and the specific nutrient requirements throughout chlamydial development. As a consequence, axenic growth of chlamydiae has not yet been achieved.

The chlamydial developmental cycle comprises two major stages: the intracellular replicative reticulate bodies (RBs) and the extracellular, non-dividing, infectious elementary bodies (EBs). Transition stages are referred to as intermediate bodies (IBs) [22]. After their uptake into a host cell EBs differentiate into RBs, which then replicate within a membrane-enclosed compartment. At the end of the infection cycle the bacteria differentiate back into EBs, which are subsequently released into the environment to infect neighboring cells [23]. Chlamydial EBs, which thus serve as dispersal stage, have been shown to differ from RBs in several structural and biochemical features, which is thought to reflect adaptation to their main biological functions of extracellular survival and reinfection [24]. Accordingly, this developmental form was reported to be more resistant to harsh conditions and mechanical stress than the fragile replicative form due to its rigid, highly cross-linked outer membrane [25], [26] that has been suggested to represent a permeability barrier inhibiting uptake of nutrients [25], [27], [28]. EBs, moreover, have a distinct ultrastructure that is characterized by highly condensed chromatin [24]. DNA compaction, achieved by the action of chlamydial histone-like proteins [29]–[32], has been proposed to cause a complete shutdown of transcriptional activity in EBs [33], [34], which is also consistent with their reduced RNA to DNA ratio compared to the replicative stage [35].

Due to these structural and biochemical features, EBs have been thought to be completely metabolically inert particles. However, this concept has been challenged by recent studies. EB proteomes of diverse chlamydial species, including members of the *Chlamydiaceae*, as well as *P. amoebophila*, have been shown to comprise remarkably complete sets of proteins involved in transcription, translation, and energy metabolism [36]–[40]. Moreover, we recently observed that the chemically defined medium DGM-21A, which had originally been developed for cultivation of *Acanthamoeba* spp. [41], [42], sustains host-free activity of the infectious stage of chlamydiae [43]. More specifically, EBs of *P. amoebophila* and *C. trachomatis* incubated in DGM-21A maintained their ability to take up the amino acid L-phenylalanine in a process that could be reversibly inhibited by an ionophore, which demonstrated that EBs are dependent on a membrane potential and are able to reenergize their membrane [43]. Most recently, sustained metabolic activity of chlamydial EBs was shown in a study that demonstrated transcription and protein biosynthesis in host-free *C. trachomatis* EBs [44].

In the current study we focused on an in-depth investigation of the metabolic potential of *P. amoebophila* EBs in order to decipher the nature and biological significance of their activities. By applying a comprehensive combination of fluorescence microscopy- and mass spectrometry-based techniques, we could demonstrate respiratory activity and D-glucose utilization in EBs and, furthermore, could obtain first insights into the host-free central carbon metabolism of *P. amoebophila*. Importantly, our investigations revealed that the availability of a metabolizable substrate during host-free incubation significantly extends maintenance of infectivity in both, *P. amoebophila* and *C. trachomatis*, indicating a biological role for metabolic activity in the infectious stage.

## Results

### Respiratory Activity in Host-Free *P. amoebophila* EBs

*P. amoebophila* developmental stages were purified from amoeba host cells and physically separated from each other by density gradient centrifugation. This approach was originally described almost 50 years ago [35], [45] and is today widely applied for the analysis of *Chlamydiaceae* developmental forms (*e.g.* [39], [44]). We optimized this purification method for *P. amoebophila* in a previous study and have quantitatively evaluated the purity of obtained EB- and RB-enriched fractions using transmission electron microscopy (TEM) [43] (Fig. S1).

Host-free activity of *P. amoebophila* was initially analyzed by using the redox dye 5-cyano-2,3-ditolyl tetrazolium chloride (CTC), which is a non-fluorescent soluble molecule that is reduced by metabolically active cells, leading to intracellular deposition of bright red fluorescent crystals [46], [47]. Due to its good correlation with other measures of cellular respiration and studies indicating an involvement of electron transport chain activity, CTC reduction is considered to be an indicator for respiratory activity [47]–[50]. Purified EBs and RBs, as well as an intermediate fraction representing a mixture of all developmental stages of *P. amoebophila*, were incubated for 2 h in host-free modified DGM-21A medium (in this study referred to as DGM-D to indicate the presence of D-glucose) containing 5 mM CTC. Prior to fluorescence microscopic analysis, bacteria were additionally stained with the DNA dye 4′,6-diamidino-2-phenylindole (DAPI). Cells containing red fluorescent crystals were observed in all fractions, irrespective of whether the assessment of activity was started directly after the purification or after a 40 h pre-incubation in DGM-D (Fig. S2A–C). Heat-inactivated bacteria were inactive, indicating that CTC reduction was not an artifact caused by constituents of the incubation medium (Fig. S2D).

In order to exclude that residual host cell components that may be present in suspensions of purified bacteria contributed to observed activities, the CTC reducing capacity of lysates of uninfected *Acanthamoeba* was assessed as control. When freshly prepared lysates were analyzed, formation of CTC crystals could also be observed in the absence of bacteria (Fig. S2E). However, these signals, which were most likely derived from host mitochondria, could be clearly distinguished from active bacteria, due to their larger size and irregular shape. In addition, they did not co-localize with bright DAPI signals that can typically be observed for living bacteria, but not for mitochondria or other components in host cell lysates, which are only weakly stained with this dye. CTC crystals of similar appearance, potentially formed by remaining host components, were occasionally observed in fractions of purified chlamydiae, but were not considered during quantitative assessments of bacterial activity. Most importantly, CTC reduction was not detected in host lysates that were pre-incubated for 40 h before analysis (Fig. S2E), demonstrating that residual host-derived activity is not stable under these conditions.

**Figure 1:**
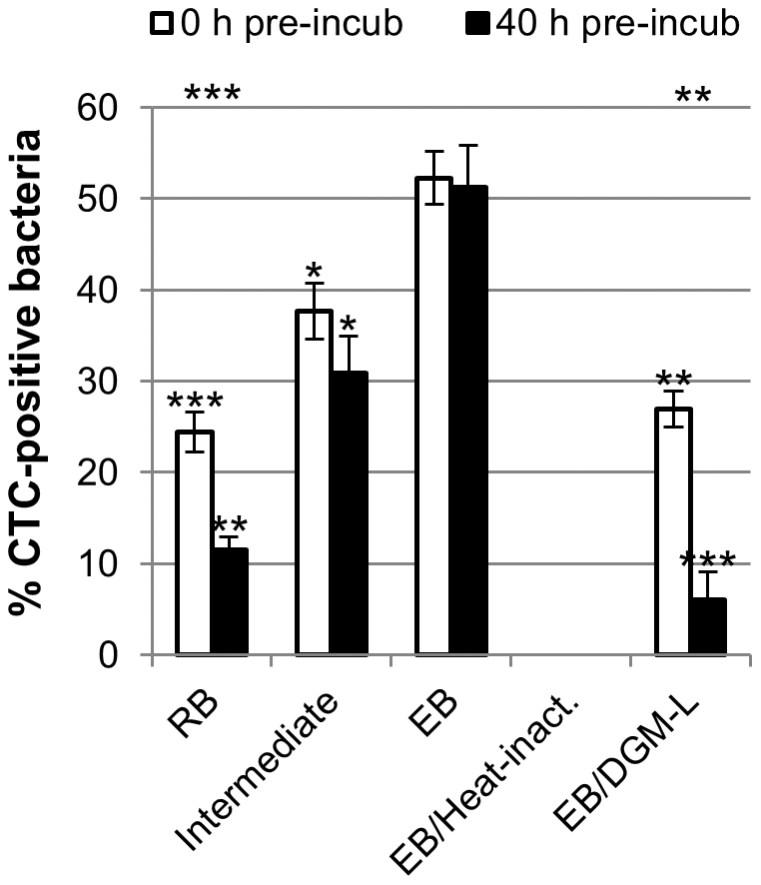
Respiratory activity of *P. amoebophila* developmental stages and effect of D-glucose deprivation. Fractions of *P. amoebophila* developmental forms were subjected to host-free incubation in DGM-D (or DGM-L, if indicated) containing 5 mM CTC, either immediately after purification (“0 h pre-incub”) or after a 40 h pre-incubation in the respective medium (“40 h pre-incub”), followed by the detection of bacteria with the DNA dye DAPI. Heat-inactivated EBs were included as control. The percentage of CTC-positive DAPI-stained bacteria was determined and subsequently corrected based on observed differences in the detectability of bacteria among fractions. Displayed data represent means and standard deviations of three independent experiments. For each experiment and condition, in total 1500 bacteria were considered for the quantification of CTC-positive bacteria, and 450 bacteria for the subsequent correction. Statistically significant differences compared to the EB fraction (ANOVA) are indicated by stars located directly above the bars, significant differences between immediate activity and activity after pre-incubation (t-test) are indicated by the stars at the upper edge of the diagram (***, p≤0.001; **, p≤0.01; *, p≤0.05).

In order to directly compare the respiratory activity between developmental forms in freshly purified suspensions, the proportion of DAPI-stained chlamydiae able to reduce CTC was determined. Examination using differential interference contrast (DIC) microscopy additionally revealed that most bacteria in EB and intermediate fractions were detectable by DAPI (90.0% (±4.1) and 79.1% (±4.0), respectively), whereas a significant proportion of bacteria in the RB fraction (49.1% (±2.7)) were not. This suggests that, consistent with the frequently reported fragility of this developmental stage [22], [35], [44], many RBs were damaged during the purification procedure or rapidly lysed during the short host-free incubation with CTC. Proportions of active bacteria were therefore corrected to account for the different detectability with DAPI. This analysis revealed that only 24.4% (±2.2) of the bacterial particles in the RB fraction were active, whereas 52.3% (±2.9) of the bacteria in the EB fraction contained intracellular CTC crystals (Fig. 1).

When bacterial activity was assessed after a host-free incubation for 40 h in DGM-D, a highly significant decrease in the proportion of active bacteria was observed in the RB fraction, in which only 11.5% (±1.4) of the DIC-detectable particles had maintained their ability to reduce CTC (Fig. 1) (t-test, p≤0.001). This observation can most likely be explained by significant lysis of the more fragile RBs during the extracellular incubation. However, the degree of bacterial disintegration might be greatly underestimated by our assessment due to the fact that completely lysed bacteria might not be detectable in DIC. Based on this consideration and the fact that, according to a recent ultrastructural analysis, suspensions of purified RBs initially still contain about 5% EBs [43], it appears most likely that this residual activity originates from the more stable EBs in this fraction. In fact, the proportion of active bacteria in the EB fraction, which initially contained about 76% mature EBs and only 8% RBs [43], was still 51.3% (±4.6) after 40 h incubation and thus almost unchanged compared to the active proportion detected in freshly purified suspensions (Fig. 1).

Collectively, these findings demonstrate that respiratory activity in EB fractions cannot be solely attributed to a contamination with other developmental stages or host-derived components, but truly occurs in *P. amoebophila* EBs. They furthermore indicate that EB activity is stable in a host-free environment, whereas activity declines more rapidly in RB fractions. This observation, together with the observed instability of host-derived activity, was exploited in the following experiments, in which a pre-incubation of purified bacteria was intentionally applied not only to ensure that truly host-free metabolic activity is assessed, but also to exclude significant contributions of co-purified RBs or host components.

### Effect of D-Glucose Deprivation on Host-Free Respiratory Activity in EBs

The observation of sustained respiratory activity in *P. amoebophila* EBs during host-free incubation suggested that the DGM-D medium contains substrates that the bacteria can utilize to fuel metabolic activities. The defined medium includes a variety of potential carbon compounds, including all proteinogenic amino acids, some of which may also be fed into energy generating metabolic pathways according to the predictions from genome annotation [14]. DGM-D, however, also contains D-glucose, and we recently observed that host-free activity of chlamydiae could not be sustained in the incubation medium reported by Hatch [43], which as a major difference to DGM-D does not contain any carbohydrates [51]. This consideration and the fact that the *P. amoebophila* genome encodes a complete pathway for D-glucose catabolism [14] prompted us to test whether D-glucose deprivation would affect host-free activity of *P. amoebophila* EBs. Complete withdrawal of glucose from DGM-D would change the physicochemical characteristics of the medium, such as the osmolarity, and it is unknown whether this could have an effect on bacterial activity. Respiratory activity of EBs was thus analyzed in a modified medium termed DGM-L, in which D-glucose was not simply omitted but replaced by its stereoisomer L-glucose, which cannot be metabolized by most organisms [52]–[54]. This replacement of substrates led to a significant decrease in the proportion of active bacteria, as inferred from their capability to reduce CTC (Fig. 1 and S2F) (Analysis of variance (ANOVA), p≤0.01). Moreover, though directly after purification from host cells 27.0% (±1.9) of the EBs detectable in DIC appeared to be metabolically active in DGM-L, a large reduction in active cells (to 6.0% (±3.1)) was observed when purified EBs were analyzed after 40 h pre-incubation in this D-glucose-free medium (Fig. 1).

These findings demonstrate that the nutrient composition of the host-free incubation medium affects *P. amoebophila* EB activity as well as its maintenance. Moreover, they suggest that D-glucose might serve as an energy source for the bacteria during host-free incubation.

### D-Glucose Uptake by Host-Free *P. amoebophila* EBs

In order to further explore the potential for D-glucose utilization by *P. amoebophila* EBs, we next investigated whether the bacteria were able to import this substrate under host-free conditions by applying 2-[*N*-(7-Nitrobenz-2-oxa-1,3-diazol-4-yl)amino]-2 deoxy-D-glucose (2-NBDG), a fluorescent analog that has previously been used as an indicator for D-glucose uptake in living cells [55]. In addition, isotope ratio mass spectrometry (IRMS), a method suitable for the analysis of the isotopic composition of biological samples [56], was applied as a supplementary technique to directly prove the import of non-derivatized sugar molecules.

**Figure 2:**
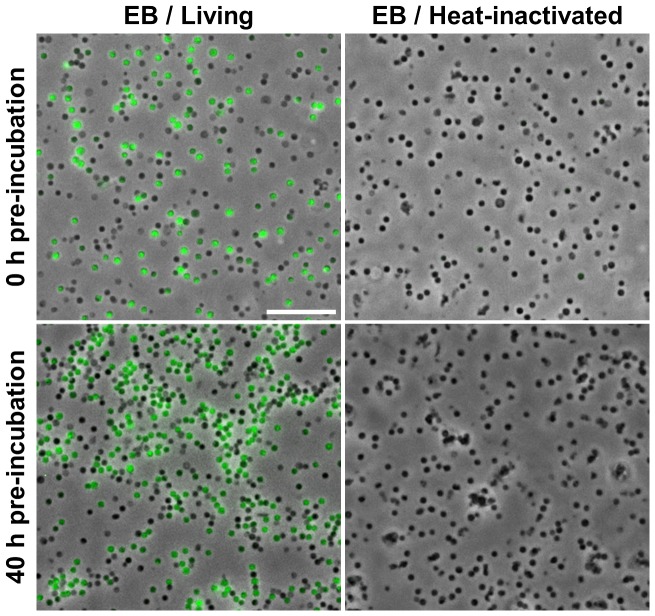
Visualization of D-glucose uptake by host-free *P. amoebophila* EBs. Living or heat-inactivated *P. amoebophila* EBs were subjected to host-free incubation in DGM-D/2 containing 100 µM of the green-fluorescent D-glucose analog 2-NBDG. The incubation was conducted either immediately after purification or after a 40 h pre-incubation in DGM-D. Representative fluorescence and DIC images are shown. The bar indicates 10 µm.

For the fluorescence-based assay, purified living and heat-inactivated EBs were incubated for 10 h with 100 µM 2-NBDG in DGM-D/2, a medium containing a reduced concentration of non-fluorescent D-glucose, followed by microscopic examination. 2-NBDG uptake could be detected in living but not in heat-inactivated bacteria, irrespective of whether they were assessed directly after purification or after pre-incubation in DGM-D. The proportion of chlamydiae able to take up 2-NBDG remained stable over time and was similar to the proportion of respiratorily active cells, comprising 47.8% (±1.7) or 53.4% (±3.4) of all bacterial particles before or after pre-incubation, respectively (Fig. 2).

For IRMS analysis, a pre-incubated EB-enriched fraction was further incubated for 48 h in DGM-D-13C, a medium in which D-glucose was replaced by its fully ^13^C-labeled isotopolog (D-[U-13C6]-glucose). Bacterial biomass was then subjected to IRMS. Based on the measured carbon content of the biomass and the ratio of ^13^C to ^12^C in the sample, the amount of ^13^C that was incorporated by purified EBs during the whole period of host-free incubation could be calculated. Although a slight enrichment in ^13^C (11.0 (±1.9) nmol ^13^C/mg dry weight (DW)) was also detected for heat-inactivated bacteria (Fig. S3), presumably as a result of substrate adsorption to the bacterial surface, a significantly higher incorporation (48.1 (±7.4) nmol ^13^C/mg DW) was observed for living bacteria (t-test, p≤0.001).

Taken together these findings clearly demonstrate active uptake of D-glucose by host-free *P. amoebophila*. The high proportion of bacteria able to import the fluorescent analog 2-NBDG once more shows that this activity is not restricted to a small proportion of possibly co-purified RBs or transition stages, but occurs in the EB stage. The consistency between the percentage of respiratorily active bacteria and bacteria that imported D-glucose further supports the concept that *P. amoebophila* EBs may not only import, but also metabolize D-glucose under host-free conditions.

### Catabolism of D-Glucose by Host-Free *P. amoebophila* EBs

Host-free incubations with D-[U-13C6]-glucose were carried out in gas-tight vials. Thus, not only incorporation of the label into the biomass, but also respiratory activity, inferred from ^13^CO_2_ release, could be analyzed. For this purpose, gas samples that were collected from the headspace of the incubations were subjected to IRMS analysis to measure the amount of CO_2_ and the atom percent ^13^C (At%^13^C) in the CO_2_. When expressed as enrichment relative to values obtained for blank incubations of bacteria-free media, these serve as measure for CO_2_ production and for the enrichment of ^13^C, respectively.

**Figure 3:**
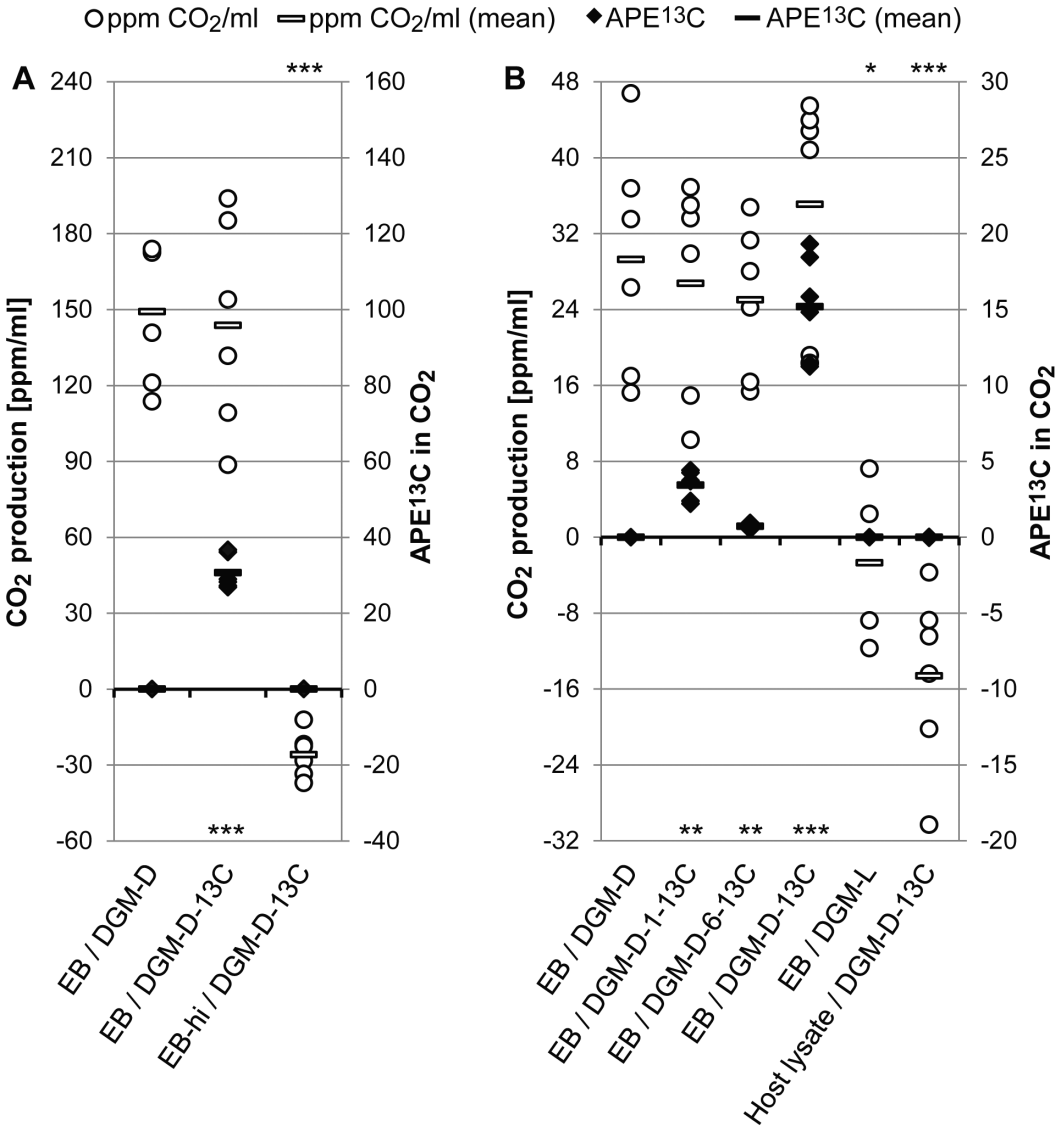
D-glucose catabolism by host-free *P. amoebophila* EBs revealed by IRMS. Purified *P. amoebophila* EBs (EB-enriched fraction (A); highly pure EB fraction (B)) were pre-incubated for 40 h in DGM-D, followed by 48 h incubation in different media (DGM-D or DGM-D-13C (A); DGM-D, DGM-D-1-13C, DGM-D-6-13C, DGM-D-13C, or DGM-L (B)) and subsequent CO_2_ measurement in the headspace of incubations by IRMS. As control, heat-inactivated bacteria (“EB-hi”) (A) or host cell lysates (B) incubated in DGM-D-13C were handled in parallel. CO_2_ production (in ppm CO_2_/ml; white circles) and the APE^13^C in the CO_2_ (black diamonds) are displayed. Diamonds and circles indicate results from individual replicates, bars display mean values. The black solid line highlights the base line for CO_2_ release and APE^13^C, which corresponds to values observed in the blanks, *i.e.* bacteria-free incubations of the respective media. Results from three independent experiments each consisting of two replicate incubations per condition are shown. An exception was the incubation in DGM-L, for which only two experiments were conducted. Statistically significant differences are indicated (for CO_2_ release and APE^13^C at the upper or lower edge of the diagram, respectively) in respect to living bacteria incubated in DGM-D (ANOVA) (***, p≤0.001; **, p≤0.01; *, p≤0.05). Note that bacterial numbers that were applied per incubation were similar between replicate experiments (between 3.9×10^9^ and 5.9×10^9^ bacteria (A); between 6.3×10^8^ and 1.0×10^9^ bacteria (B)), but were significantly different between (A) and (B), explaining observed differences in the extents of CO_2_ production.

In general, CO_2_ amounts in the headspace of incubations were increased compared to the composition of normal lab air even in the absence of bacteria, suggesting that a part of the newly formed CO_2_ may be derived from outgassing carbonate. However, the presence of living bacteria led to a marked increase of 146.5 (±33.9) ppm CO_2_/ml (Fig. 3A). Moreover, a significant enrichment of ^13^C in CO_2_ (atom percent ^13^C enrichment (APE^13^C) 30.7 (±4.5)) could be detected in the presence of D-[U-13C6]-glucose (ANOVA, p≤0.001). In contrast, biological CO_2_ production was not observed when heat-inactivated bacteria were used.

Taken together, these findings provide further evidence that EBs of *P. amoebophila* are metabolically active under host-free conditions and demonstrate that the observed CO_2_ formation can, at least partly, be attributed to D-glucose catabolism.

### Insights into D-Glucose Metabolism in Host-Free *P. amoebophila* EBs by IRMS

Though CO_2_ production evidences metabolic activity it does not necessarily indicate complete catabolism of a given substrate, as several biochemical pathways involve only partial substrate breakdown. Defining the exact position of carbon atoms in the substrate molecule that are liberated as CO_2_ thus aids in a better understanding of the activity of certain metabolic pathways. In the present study, the contribution of different carbon atoms in D-glucose to CO_2_ produced by host-free EB activity was investigated by IRMS. For this purpose, a pre-incubated EB fraction was further incubated for 48 h in DGM-D, DGM-D-13C, DGM-D-1-13C, or DGM-D-6-13C. These media contained unlabeled D-glucose, fully ^13^C-labeled D-glucose (D-[U-13C6]-glucose), D-glucose labeled exclusively at carbon 1 (D-[1-13C]-glucose), or D-glucose labeled at carbon 6 (D-[6-13C]-glucose), respectively. Moreover, incubation of EBs in DGM-L was included to analyze the effect of D-glucose deprivation on CO_2_ production. Host cell lysates incubated in DGM-D-13C were handled in parallel to test for potential contributions of residual host-derived activity.

CO_2_ production was observed for all incubations of living bacteria in media containing D-glucose, as inferred from an average increase in detectable CO_2_ of 29.0 (±11.1) ppm/ml relative to amounts detected in blank incubations of bacteria-free media (Fig. 3B). Taking into account that about six times fewer bacterial cells were applied per incubation, the extent of CO_2_ release appeared to be very similar to that observed in the previous experiment (Fig. 3A). Substrate deprivation, *i.e.* the replacement of D-glucose by L-glucose, reduced CO_2_ formation to background levels (Fig. 3B). Respiratory activity was also not observed in amoebal lysates. An enrichment of ^13^C in CO_2_ was detected for all incubations of living bacteria in media containing ^13^C-labeled D-glucose. However, the degree of labeling was highly variable depending on the variant of labeled substrate included. As expected the greatest formation of ^13^CO_2_ was observed in the presence of the fully labeled D-glucose (APE^13^C 15.2 (±3.4)), which was about 4.4 times higher than that observed for D-[1-13C]-glucose and about 21.2 times higher than that observed for D-[6-13C]-glucose. The calculated ratio for the APE^13^C was 21.2 (DGM-D-13C):4.8 (DGM-D-1-13C):1.0 (DGM-D-6-13C). Production of ^13^CO_2_ could not be detected for host cell lysates.

Liberation of carbon 6 from D-glucose as CO_2_ is usually only observed as a consequence of tricarboxylic acid (TCA) cycle activity and is therefore evidence for a functional sugar catabolism in extracellular EBs of *P. amoebophila*. However, our data do not support a scenario in which all assimilated D-glucose would be fully catabolized to CO_2_ via glycolysis and/or pentose phosphate pathway (PPP) combined with TCA cycle activity, as this would result in equal amounts of released carbon 1 and carbon 6. The preferred release of carbon 1 indicates that D-glucose also passes through the oxidative part of the PPP without being coupled to a subsequent complete breakdown of its products. Moreover, the observed extent of ^13^CO_2_ production from fully labeled D-glucose additionally indicates that also carbon atoms other than carbon 1 are released from D-glucose as CO_2_ in metabolic reactions that do not lead to complete catabolism. Thus, a proportion of the metabolized D-glucose may be devoted to anabolic reactions, including for example pathways such as fatty acid and isoprenoid synthesis, which begin with a decarboxylation of pyruvate. We focused on the three metabolic scenarios outlined above to model their contributions to CO_2_ production using the calculation described in detail in Fig. S4. Our data would be consistent with a theoretical ratio of 6.2 (putative anabolic reactions):3.8 (oxidative part of PPP):1.0 (complete catabolism). This finding suggests that the experimentally obtained data are plausible and can be explained by a reasonable simplified metabolic model. The calculation, furthermore, suggests that a major proportion of D-glucose is not completely degraded, but may enter anabolic pathways.

### Central Carbon Metabolism of Host-Free *P. amoebophila* EBs Revealed by Mass Spectrometric Metabolite Analysis

In order to obtain deeper insights into the metabolism of host-free *P. amoebophila* EBs and to explore the intracellular fate of ^13^C-labeled D-glucose we next conducted a mass spectrometric metabolite analysis combining ion cyclotron resonance Fourier transform mass spectrometry (ICR/FT-MS) and ultra-performance liquid chromatography mass spectrometry (UPLC-MS). ICR/FT-MS offers ultra-high resolution, enabling the distinction of several thousands of ions. This technique provides extremely high mass accuracy, which allows direct calculation of the elemental composition of detected compounds and thereby facilitates metabolite annotation and clear discrimination between isotopologs [57], [58]. However, as chromatographic separation of analytes prior to mass spectrometric analysis carries several advantages over direct injection experiments such as decreased matrix effects, separation of isobaric compounds, and delivery of additional information about physicochemical properties of analytes by their retention time [59], [60], we additionally conducted UPLC-MS as second analytical technique, in particular to verify the detection of labeled metabolic intermediates.

Prior to the analysis, metabolites were extracted from an EB-enriched fraction that had been pre-incubated and subsequently incubated for 48 h in DGM-D-13C15N, a modified medium containing ^13^C-labeled D-glucose and ^13^C^15^N-labeled L-phenylalanine, or in DGM-D, containing the corresponding unlabeled substrates. Heat-inactivated bacteria incubated in DGM-D-13C15N were also analyzed. Labeled L-phenylalanine was included in this experiment as a control, as it is a substrate that is taken up by host-free EBs [43] and which exhibits only limited potential of being further metabolized by *P. amoebophila* according to predictions from the genome [14].

**Figure 4:**
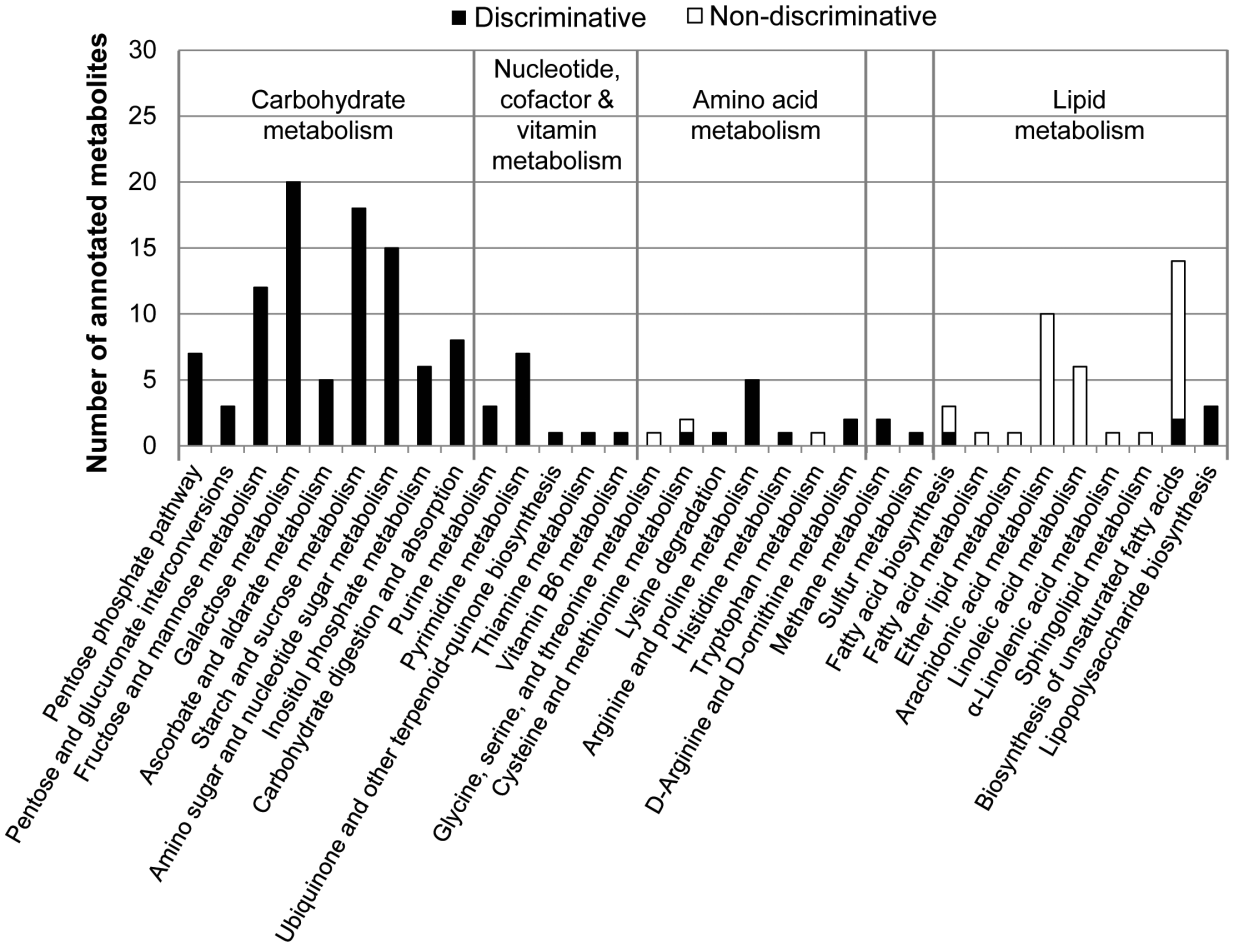
Overview of ICR/FT-MS-detected annotated compounds and of metabolites discriminative for living compared to inactivated EBs. An EB-enriched fraction of *P. amoebophila* was pre-incubated for 40 h in DGM-D, followed by 48 h incubation in DGM-D or DGM-D-13C15N and subsequent mass spectrometric analysis of metabolite extracts. Heat-inactivated EBs incubated in DGM-D-13C15N were included as control. M/z features detected by ICR/FT-MS in samples of DGM-D-incubated EBs were annotated by MassTRIX [61], followed by data analysis using PLS-DA (Fig. S6) to extract the most discriminative compounds characterizing living compared to inactivated EBs. The PLS-DA model included data from DGM-D-incubated living and DGM-D-13C15N-incubated inactivated EBs. Bars indicate the total number of annotated metabolites that were assigned to specific KEGG pathways. The number of metabolites that were found to be discriminative (black) or non-discriminative (white) for living EBs are additionally indicated. Note that metabolites from carbohydrate, nucleotide, cofactor, vitamin, and amino acid metabolism were more abundant in living EBs, whereas the pattern of lipid species was more similar between living and inactivated bacteria.

ICR/FT-MS analysis enabled the assignment of 1674 and 1767 m/z features to DGM-D-13C15N- or DGM-D-incubated bacteria, respectively. In order to gain a first impression on the sample composition, masses that were detected in extracts from DGM-D-incubated EBs were submitted to MassTRIX for annotation [61]. This analysis revealed that metabolites from several different biochemical pathways were detected; including compounds involved in amino acid-, nucleotide-, sugar-, and lipid metabolism (Fig. 4). Spectra of living bacteria were highly similar to each other irrespective of the applied incubation medium, indicating that the presence of the stable isotope-labeled substrates did not affect the metabolite pattern (Fig. S5A). To verify this visual impression, we investigated the data using principal component analysis (PCA). Extracts from living bacteria that were incubated either in DGM-D-13C15N or in DGM-D clustered together in the PCA ordination, which confirms that the samples were comparable in their general composition (Fig. S5B). We additionally produced PCA ordinations including data from the heat-inactivated EBs. Both ordinations, which included dead bacteria incubated in DGM-D-13C15N and living bacteria incubated either in DGM-D or in DGM-D-13C15N, revealed a separation between living and inactivated bacteria that reflects the presence of different molecular patterns (Fig. S5C–D). Metabolites that are discriminative for living EBs, *i.e.* compounds that can be detected in living, but not or only in low amounts, in inactivated bacteria, might represent valuable indicators for an active metabolism. As a next step we applied a partial least square discriminative analysis (PLS-DA) model including data from DGM-D-incubated living and DGM-D-13C15N-incubated inactivated EBs (R^2^Y(cum) = 0.926,Q^2^ = 0.834) (Fig. S6). MassTRIX annotation of the most relevant m/z features revealed that metabolites from the amino acid-, nucleotide-, and central carbon metabolism were discriminative for living EBs, whereas the pattern of metabolites assigned to lipid metabolism appeared to be less affected (Fig. 4).

**Figure 5:**
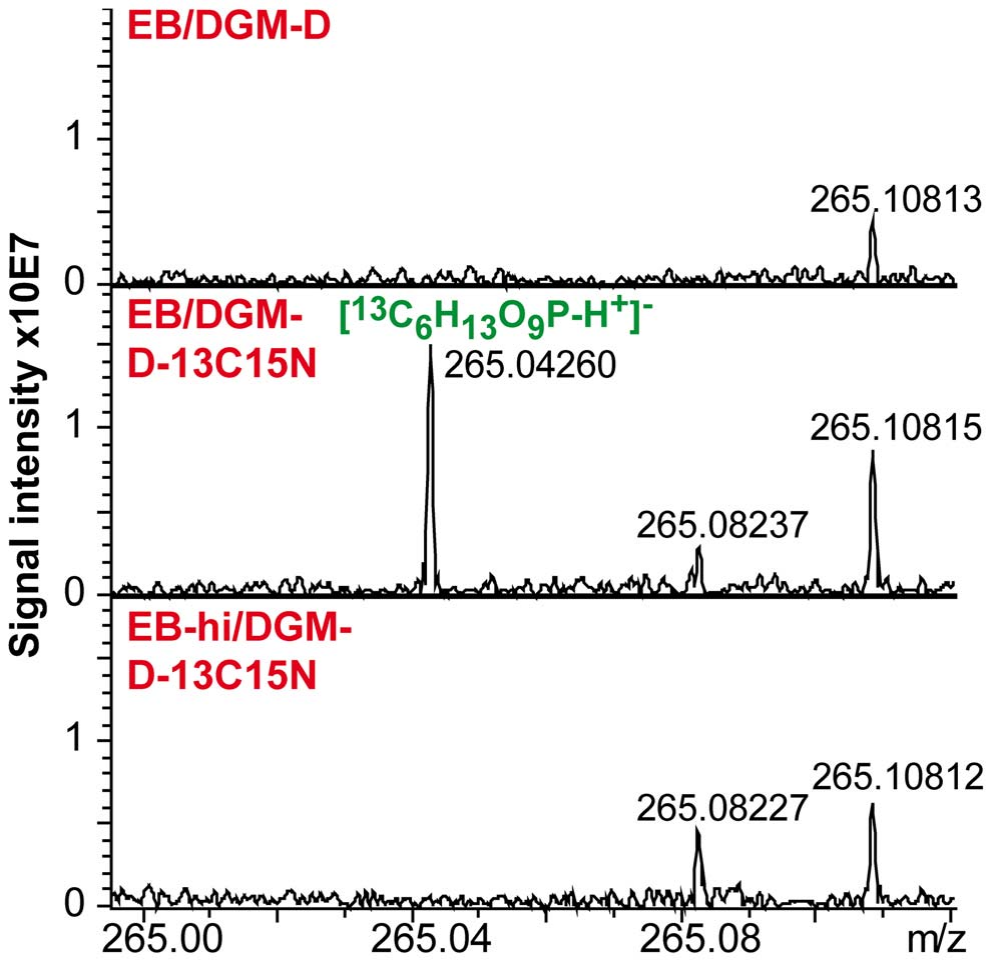
ESI(-)ICR/FT-MS spectra indicating host-free synthesis of hexose-P by *P.* **amoebophila**** **EBs.** **** An enlarged view on the mass range 265.00–265.12 in ESI(-)ICR/FT-MS spectra of DGM-D- and DGM-D-13C15N-incubated living and DGM-D-13C15N-incubated inactivated bacteria is shown. Note that the peak indicating fully ^13^C-labeled hexose-P (^13^C_6_H_13_O_9_P) can only be seen in spectra from DGM-D-13C15N-incubated living EBs, but not in the controls, demonstrating host-free synthesis of hexose-P by *P. amoebophila* EBs.

To obtain deeper insights into the central carbon metabolism of host-free EBs, further data analysis focused on ^13^C-labeled metabolites that were derived from ^13^C-labeled D-glucose. Labeled metabolites were identified based on their accurate mass and presence of their unlabeled metabolite analogs in extracts of bacteria incubated in DGM-D by application of mass difference-based networks. Peaks corresponding to fully labeled glucose (C_6_H_12_O_6_) and phenylalanine (C_9_H_11_NO_2_) could be readily detected in extracts from bacteria incubated in DGM-D-13C15N (Table 1). Both metabolites were, however, also detected in ICR/FT-MS and UPLC-MS spectra from heat-inactivated bacteria. This suggests adsorption of these substrates to bacterial surfaces, a phenomenon that was already noted in the IRMS-based analysis (Fig. S3). Beside glucose and phenylalanine, 34 additional fully or partially ^13^C-labeled metabolites were detected in ICR/FT-MS spectra of living, but not heat-inactivated, bacteria incubated in DGM-D-13C15N (Table 1 and S1). According to their predicted chemical composition, some of these metabolites could be annotated as phosphorylated hexose (C_6_H_13_O_9_P, hexose-P) (Fig. 5), biphosphorylated hexose (C_6_H_14_O_12_P_2_, hexose-PP), a short chain hydroxy acid (C_6_H_12_O_7_, *e.g.* gluconate), a phosphorylated heptose (C_7_H_15_O_10_P, heptose-P), a short chain tricarboxylic acid (C_6_H_8_O_7_; *e.g.* citrate), a disaccharide (C_12_H_22_O_11_), and a trisaccharide (C_18_H_32_O_16_). UPLC-MS analysis confirmed the presence of these metabolites. Only the detection of biphosphorylated hexose could not be verified, due to the absence of the corresponding peak of the labeled and unlabeled compound in UPLC-MS spectra. Moreover, UPLC-MS detected several additional labeled metabolites, such as phosphorylated pentose (C_5_H_11_O_8_P, pentose-P), phosphorylated tetrose (C_4_H_9_O_7_P, tetrose-P), and a short chain acyl phosphate (C_3_H_5_O_6_P; *e.g*. phosphoenolpyruvate) (Table 1).

**Table 1:** Annotated ^13^C-labeled metabolites detected in DGM-D-13C15N-incubated EBs by a combination of ICR/FT-MS and UPLC-MS.

		Detected m/za)			
Elemental composition	Annotation (example)	[M-H^+^]^−^	[M+Cl^−^]^−^	Number of ^13^C atomsb)	Detection methodc)
C_6_H_12_O_6_	Glucose	185.07624	221.05292	6d)	A(H,Cl); B(H,Cl)
		184.07288	220.04957	5d)	A(H,Cl); B(H,Cl)
C_6_H_13_O_9_P	Hexose-P	265.04257		6	A(H); B(H)
		264.03923		5	A(H); B(H)
		262.03246		3	A(H); B(H) – traces
C_6_H_14_O_12_P_2_	Hexose-PP		377.97560	3	A(Cl)
C_3_H_5_O_6_P	Phosphoenol-pyruvate		205.961	3	B(Cl)
C_6_H_8_O_7_	Citrate	197.040		6	B(H)
		196.037		5	B(H)
		195.03315		4	A(H); B(H)
		194.02979		3	A(H); B(H)
		193.026		2	B(H)
C_5_H_11_O_8_P	Pentose-P	234.029		5	B(H)
C_7_H_15_O_10_P	Heptose-P	296.05649		7	A(H); B(H)
		295.05314		6	A(H); B(H)
C_4_H_9_O_7_P	Tetrose-P	203.015		4	B(H)
		202.011		3	B(H)
C_6_H_12_O_7_	Gluconate	201.07116		6	A(H)
C_12_H_22_O_11_	Disaccharide	353.14920	389.12587	12d)	A(H,Cl); B(H,Cl)
		352.146	388.12252	11d)	A(Cl); B(H)
			383.10575	6	A(Cl)
C_18_H_32_O_16_	Trisaccharide		557.19883	18	A(Cl) – traces

**^a)^**The detected m/z is given for each ion species with the instrument given accuracy.

**^b)^**Labeled metabolites were considered to be present in extracts of DGM-D-13C15N-incubated EBs when peaks corresponding to the exact mass of the unlabeled metabolites were detected in the DGM-D-incubated controls and observed mass shifts were consistent with the exchange of ^12^C by ^13^C atoms. Metabolites containing single ^13^C atoms were also observed in extracts from DGM-D-incubated EBs, due to the natural isotopic distribution of carbon, and were thus excluded.

**^c)^**The detection method (A: ICR/FT-MS; B: UPLC-MS) is indicated, as well as the ion species detected (H: [M-H^+^]^−^; Cl: [M+Cl^−^]^−^).

**^d)^**These metabolites were detected by ICR/FT-MS analysis in purchased D-[U-13C6]-glucose and were thus not considered as host-free synthesized metabolites.

**Figure 6:**
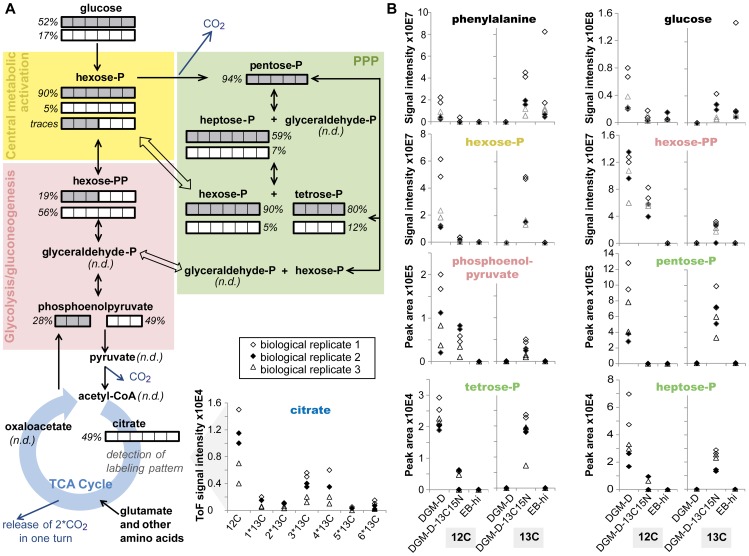
Central carbon metabolism in *P. amoebophila* EBs deduced from mass spectrometry-based metabolite analysis. A schematic representation of the central carbon metabolism in *P. amoebophila* is shown in (A). ^13^C-labeled metabolites detected by ICR/FT-MS or UPLC-MS in extracts of DGM-D-13C15N-incubated living bacteria are indicated. The isotopologs that were observed are additionally specified by bars, consisting of a number of units equal to the number of C atoms in the molecules, whereby each unit of the bar indicates either a ^12^C (white) or a ^13^C (gray) atom. Percentage values next to the bars denote the relative abundance of the isotopologs calculated from the mass signal intensity or peak area of the respective peak (for ICR/FT-MS or UPLC-MS data, respectively) compared to peaks of the unlabeled metabolite detected in DGM-D-incubated bacteria. For citrate, for which all possible isotopologs were detected by UPLC-MS, instead of bars the complete isotopolog profile observed in DGM-D-13C15N-incubated living EBs is shown. In (B), mass signal intensities and peak areas (for ICR/FT-MS or UPLC-MS data, respectively) of selected fully ^13^C-labeled (“13C”) and corresponding unlabeled (“12C”) metabolites are displayed for DGM-D-13C15N-incubated living bacteria and the controls. Note the absence of fully ^13^C-labeled isotopologs in extracts from inactivated bacteria and the appearance of labeled intermediates and the concomitant reduction in the amount of the corresponding unlabeled metabolite in samples of DGM-D-1315N-incubated living bacteria compared to DGM-D-incubated bacteria. Exceptions were the detection of labeled glucose and phenylalanine in extracts of inactivated bacteria, presumably due to substrate adsorption to the surface of the bacterial cells.

Altogether, detection of these labeled metabolites indicates synthesis of key intermediates of glycolysis/gluconeogenesis, PPP, and the TCA cycle in host-free *P. amoebophila* EBs (Fig. 6). Beside the detection of the ^13^C-labeled isotopologs, most of those metabolites were also detected as unlabeled molecules in extracts from DGM-D-13C15N-incubated EBs, indicating co-utilization of D-[U-13C6]-glucose with additional unlabeled carbon compounds. The observed abundance ratios of ^12^C and ^13^C atoms in the detected labeled metabolites suggest a rather minor glycolytic catabolic activity in host-free EBs and a predominant catabolism of D-glucose by the PPP. In fact, whereas labeled metabolites in the PPP were only detected as fully labeled molecules, clearly demonstrating their origin from D-[U-13C6]-glucose, the mass signal corresponding to biphosphorylated hexoses only indicated the presence of a partially labeled metabolite, which is inconsistent with its generation by glycolytic breakdown of fully labeled D-glucose, but may result from joining of one unlabeled C3 and one fully labeled C3 body by a reverse aldolase reaction during gluconeogenesis. This interpretation is consistent with the detection of traces of partially labeled phosphorylated hexose. In addition, the observed synthesis of a partially labeled disaccharide and traces of a labeled trisaccharide further support the occurrence of anabolic activities in host-free *P. amoebophila* EBs.

Taken together, the ICR/FT-MS and UPLC-MS analyses were fully consistent with the results obtained by IRMS measurements; they provide first detailed information on the central carbon metabolism in host-free *P. amoebophila* EBs and show the occurrence of both catabolic as well as anabolic reactions.

### Influence of Nutrient Availability on Maintenance of *P. amoebophila* Infectivity

To further explore the biological relevance of the host-free metabolic activity in EBs, which represent the infectious non-replicative developmental stage of chlamydiae [24], we analyzed the effect of nutrient availability on *P. amoebophila* infectivity. For this purpose host-free incubations were conducted in different media, including DGM-D, the modified medium in which D-glucose was replaced by L-glucose (DGM-L), a medium containing both D- and L-glucose (DGM-DL), as well as a nutrient-free buffer (phosphate-buffered saline (PBS)) or salt solution (0.6% NaCl solution) of similar pH and osmolarity. The capability of incubated bacteria to infect amoebae was then assessed at selected time points during a period of one week.

**Figure 7:**
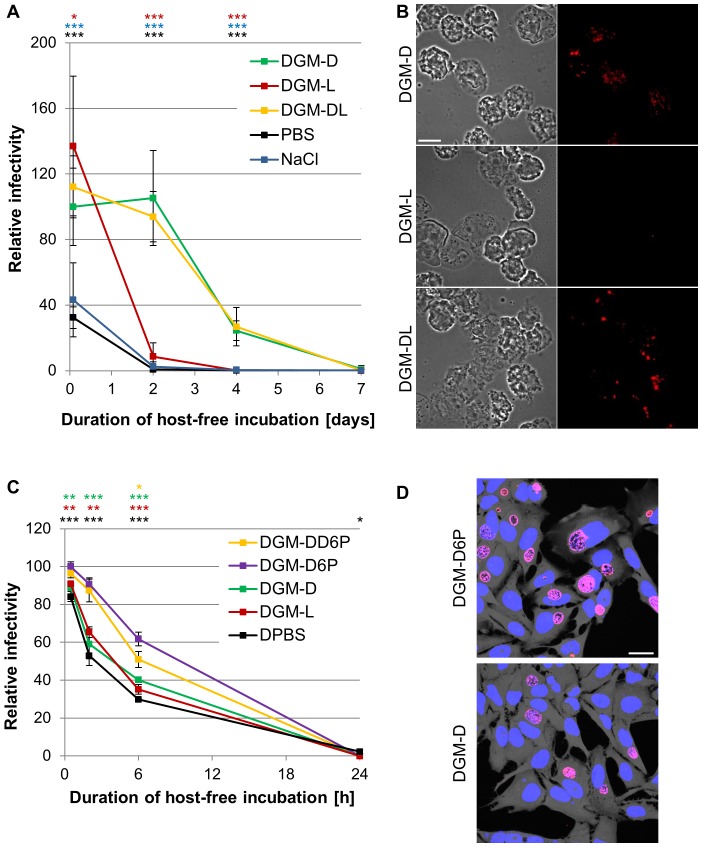
Effect of substrate availability on maintenance of infectivity. *P. amoebophila* and *C. trachomatis* cells were harvested from infected amoeba and HeLa 229 cell cultures, respectively, and incubated for indicated periods of time in different host-free media. Subsequently, incubated bacteria were used to infect amoebae (*P. amoebophila*) or HeLa 229 cells (*C. trachomatis*), which were then fixed at 48 h or 24 h p.i., respectively. Bacteria were detected by FISH (*P. amoebophila*) or immunostaining (*C. trachomatis*). The observed infectivity, relative to that observed for 2 h incubation in DGM-D (*P. amoebophila*) or 30 min incubation in DGM-D6P (*C. trachomatis*) is depicted in (A) and (C), respectively. Data represent means and standard deviations from at least three replicate host-free incubations. For each sample a minimum of 600 amoebae (A) or 300 HeLa 229 cells (C) was counted. Statistically significant differences compared to the values obtained for DGM-D (A) or DGM-D6P (C) are indicated (ANOVA; ***, p≤0.001; **, p≤0.01; *, p≤0.05). In (B) representative fluorescence and DIC images of amoebae infected with *P. amoebophila* after 48 h host-free incubation in the indicated media are shown (FISH, red). The bar indicates 10 µm. In (D) representative confocal fluorescence images of HeLa 229 cells infected with *C. trachomatis* after 2 h host-free incubation in the indicated media are shown. Bacteria were detected by immunostaining (red), host cells and DNA were stained using HCS cytoplasmic stain (grey) and DAPI (blue), respectively. The bar indicates 25 µm.

Overall, numbers of infectious particles appeared to decline over time in all tested media (Fig. 7A). In fact, under the applied incubation and infection conditions virtually no infection capability was left after a host-free period of 7 days. Initial infectivity, assessed after 2 h incubation, was similar in DGM-D and DGM-L. However, while it remained stable for the first two days of host-free incubation in DGM-D, a rapid reduction to 8.6% (±8.3) infectivity (relative to the initial value in DGM-D) was observed for bacteria incubated for this period of time in DGM-L (Fig. 7A–B) (p≤0.001; ANOVA). This finding cannot be explained by a potential toxicity of L-glucose, as incubation in DGM-DL, a medium containing both sugar stereoisomers, revealed a similar infectivity curve than observed for DGM-D (Fig. 7A). A rapid decline in the number of infectious particles was also observed in PBS and 0.6% NaCl solution. We also noted that in these nutrient-free media the initial infectivity after 2 h incubation was already significantly reduced compared to that of bacteria incubated in DGM-D (p≤0.001; ANOVA). This finding suggests that despite having a similar pH and osmolarity these media may also lack other essential components or differ in physicochemical properties required for chlamydial survival or stability. In order to exclude that D-glucose plays a role in infection beyond fueling metabolic reactions, we also assessed whether addition of D-glucose to bacteria that were incubated in DGM-L and thus starved could restore their infection capacity. However, this treatment did not affect the rapid decline of infectivity in DGM-L (Fig. S7), indicating that the supportive effect of D-glucose cannot be explained by a potential promotion of bacterial attachment or entry into host cells that might be mediated by sugar molecules adsorbed to the surface of EBs.

Collectively, these findings demonstrate that the availability of D-glucose in the host-free environment is critical for *P. amoebophila* EBs as it significantly extends maintenance of their major biological role, which is the capacity to successfully infect new host cells and to initiate a new round of intracellular replication.

### Influence of Nutrient Availability on Maintenance of *C. trachomatis* Infectivity

In order to explore whether a dependency of host-free EBs on nutrient availability can be observed for the pathogenic *Chlamydiaceae*, we compared maintenance of *C. trachomatis* (serovar L2) infectivity in different media. *C. trachomatis*, in contrast to *P. amoebophila*, lacks a gene encoding a glucokinase [12], [14]. This chlamydial species is thus unable to utilize D-glucose directly and is expected to rely on the phosphorylated derivative instead. The media used for host-free incubation of *C. trachomatis* consequently included nutrient-free Dulbecco's phosphate-buffered saline (DPBS), DGM-D, and DGM-L, as well as DGM containing D-glucose-6-phosphate instead of D-glucose (DGM-D6P) or containing both D-glucose and D-glucose-6-phosphate (DGM-DD6P). Assessment of infectivity for HeLa 229 cells conducted 30 minutes after suspension of purified bacteria in the respective incubation media indicated that already after this short exposure infectivity was significantly decreased in all media devoid of D-glucose-6-phosphate compared to DGM-D6P or DGM-DD6P (p≤0.01; ANOVA) (Fig. 7C). In addition, while infectivity remained relatively stable in DGM-D6P (90.8%±4.1) and DGM-DD6P (87.3%±7.5) during the first 2 h of host-free incubation, a larger reduction was observed in media lacking D-glucose-6-phosphate (DGM-D (59.1%±10.0), DGM-L (65.4%±3.5), and DPBS (53.0%±6.6)) (Fig. 7C–D). Surprisingly, infectivity was almost completely lost after 24 h host-free incubation in all tested media, which contrasts strongly to the prolonged extracellular survival of *P. amoebophila*.

These findings demonstrate that a dependency of infectivity maintenance on the availability of a metabolizable substrate is not restricted to the amoeba symbiont *P. amoebophila*, but can also be observed for the human pathogen *C. trachomatis*. This suggests that sustained metabolic activity in EBs may represent a more general and important feature of chlamydial biology.

## Discussion

The chlamydial EB has long been regarded as a completely metabolically inert particle that can only be reactivated after entry into a suitable eukaryotic host cell. This notion has recently been challenged [43], [44] and the data presented in the present study confirm and greatly extend the concept of metabolic activity in the infective stage of chlamydiae. Our findings demonstrate that host-free EBs of *P. amoebophila* at least temporarily interact with their environment and maintain both catabolic as well as anabolic activities. Furthermore, these activities are of major biological relevance as they contribute to prolonged survival of infectious EBs.

The purification of chlamydial developmental forms and hence also a direct investigation of their biological properties, is a challenging task. A perfect enrichment and a complete removal of transition forms, some of which may be close to RBs or EBs but not yet fully differentiated, cannot be achieved [35], [43], [62], [63] nor can a contamination with host proteins be avoided completely [36]–[40]. Nevertheless, density gradient centrifugation and TEM, as used in the present study, currently represent the most powerful approach for the separation of and discrimination between chlamydial developmental stages and has enabled important insights into the biology of *Chlamydiaceae* RBs and EBs [22], [39], [44], [64]. By taking into account the purity of *P. amoebophila* EB and RB fractions defined by TEM (Fig. S1) [43] in combination with the application of single-cell based assays (Fig. 1, 2, and S2), our data clearly allow us to attribute metabolic activity to the EB stage for the reasons outlined below.

Several lines of evidence demonstrate that the observations reported in this study cannot be explained by residual host-derived activities: (i) respiratory activity, inferred from CTC reduction, was detectable in individual bacteria and could be clearly distinguished from the short-lived activity in lysates of uninfected host cells that was lost completely after pre-incubation (Fig. 1 and S2); (ii) import of 2-NBDG, as indicator for D-glucose uptake, could be detected at the single-cell level (Fig. 2); and (iii) D-glucose catabolism, as inferred from the release of ^13^CO_2_ from ^13^C-labeled D-glucose, could be observed in pre-incubated purified EBs, whereas no CO_2_ production was detected in equally treated highly concentrated host cell lysates (Fig. 3B).

Our data also clearly show that metabolic activity cannot be solely explained by co-purified RBs or transition stages, as CTC reduction and 2-NBDG uptake were detectable in about 50% of all bacterial cells (Fig. 1 and 2) in a highly enriched EB fraction that consists of about 76% mature EBs (defined as bacteria containing only electron-dense and electron-lucent material). The high proportion of inactive bacteria may be explained by the presence of dead bacteria. This would be consistent with previous observations that revealed that directly after harvesting of bacteria from infected amoeba cultures a significant proportion of the bacteria could be stained with the membrane-impermeable DNA dye propidium iodide, indicating that they had lost their membrane integrity [43]. Consistent with reports on *Chlamydiaceae* [22], [35], [44], in particular the RBs of *P. amoebophila* appeared to be very fragile, and hence clear differences in the host-free maintenance of respiratory activity were observed between developmental stages of *P. amoebophila*. Whereas EBs maintained their ability to reduce CTC during a 40 h incubation period, the proportion of active bacteria in the RB fraction dropped to a level similar to the expected proportion of co-purified EBs (Fig. 1). We therefore concluded that RB activity was essentially lost during this 40 h incubation. Based on this observation we exploited the instability of RBs and host-derived activity by including an initial 40 h host-free incubation step as pre-treatment of EB fractions prior to the detailed characterization of their central carbon metabolism. Alternative pre-treatment procedures, which have been applied by investigators in earlier studies on host-free activities of *Chlamydiaceae* – such as protease treatment to remove host cell-derived enzymes [65]–[67], sonication [28], or the addition of detergents to cause lysis of the more fragile RBs [68], [69] – were avoided in the present study due to potential detrimental effects on bacterial surface proteins or EB viability.

Altogether, the findings presented in this study clearly demonstrate metabolic activity in *P. amoebophila* EBs and thus strongly support recent appeals to revise the dogma of the metabolic inertness of the infective stage of chlamydiae [43], [44]. New methodological approaches, differences in media composition and purification and pre-treatment of EBs before assessment of activity might explain conflicting observations in previous studies [51], [68]–[70]. Indeed, the possibility of sustained activities in EBs, although not generally recognized by the scientific community, has already been indicated in a few earlier studies. For example, it has been shown that nucleoid decondensation during the redifferentiation of *C. trachomatis* EBs to RBs shortly after their uptake into host cells depends on bacterial *de novo* transcription and translation, implying that the capability of a certain level of activity must be maintained [71]. Consistently, Sarov and Becker could show earlier that purified EBs of *C. trachomatis* were able to synthesize RNA [72], and our previous findings suggested *de novo* protein synthesis in host-free *C. trachomatis* and *P. amoebophila* EBs [43]. Both findings could very recently be confirmed for *C. trachomatis* [44]. In the context of carbon metabolism, early investigations of host-free *Chlamydiaceae* also indicated that activities detected in mixed suspensions of RBs and EBs, but so far mainly attributed to the replicative stage, remained stable for at least two days [66], [73]. This finding appears to be inconsistent with the known fragility of RBs and thus may provide further evidence for metabolic activity in the infective stage. A biological role for metabolic activities in the EB stage was furthermore indicated by a direct comparison of the protein complements of *C. trachomatis* EBs and RBs, which demonstrated that proteins required for the central metabolism and glucose catabolism were, in fact, even predominant in the infective stage [39].

**Figure 8:**
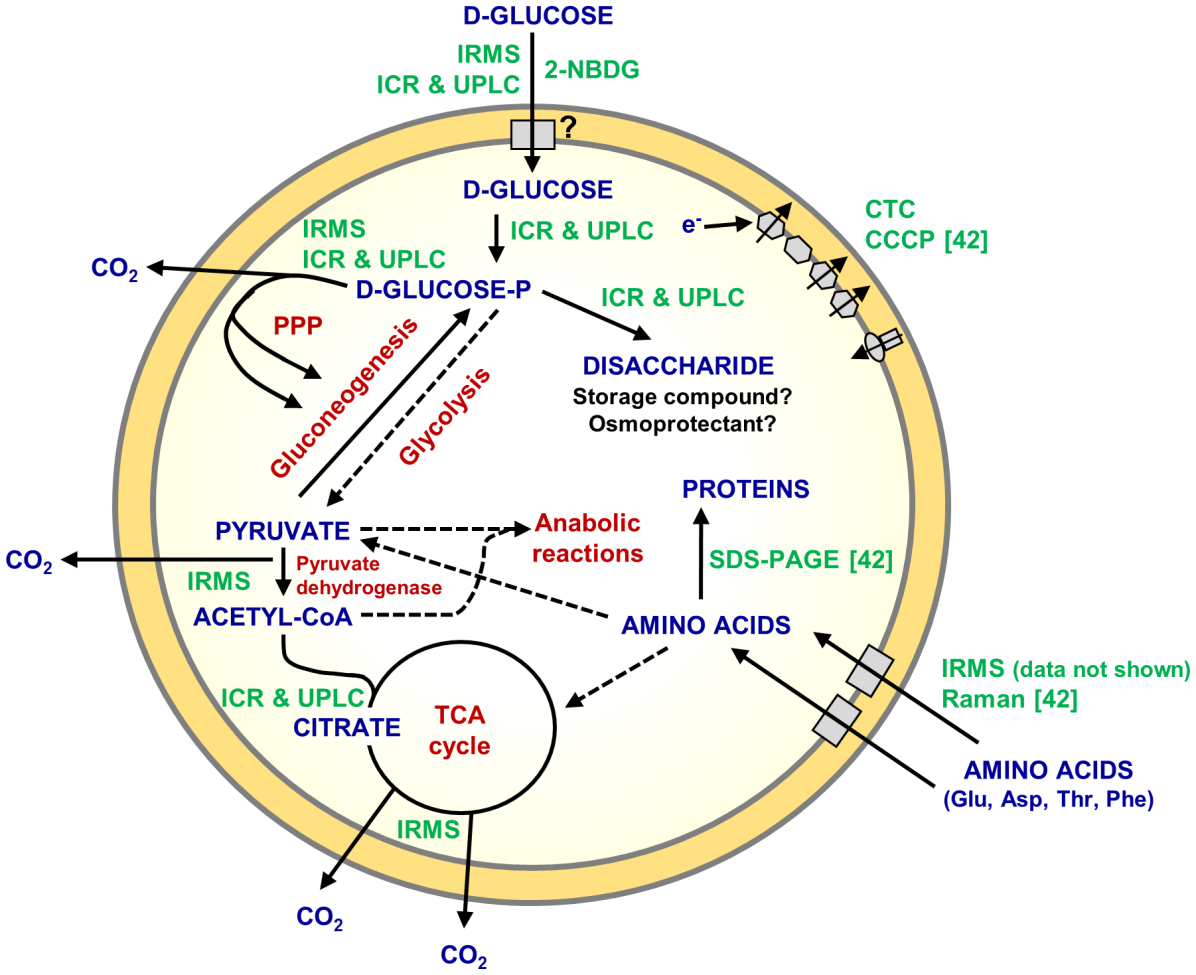
Schematic representation of host-free activity of *P. amoebophila* EBs. A metabolic model based on current knowledge is shown. The representation integrates observations from our previous investigations [43] and new findings obtained in the current study. Metabolic pathways and enzymes are illustrated in red, detected and postulated metabolites in blue. The techniques that provided experimental support for the indicated activities are shown in green (“ICR & UPLC” denotes activities confirmed by both, ICR/FT-MS and UPLC-MS). Dotted lines indicate metabolic reactions, whose occurrence in EBs was suggested, but not demonstrated. The electron transport chain, amino acid transporters, as well as a putative D-glucose importer, are depicted in the bacterial membrane as gray boxes.

In addition to these conceptual considerations, the present study represents a hitherto unmatched detailed metabolomic analysis of a member of the *Chlamydiae*. It provides invaluable insights into the central carbon metabolism of *P. amoebophila* (Fig. 8) and indicates both similarities as well as major differences to observations reported for the *Chlamydiaceae*. While it was initially proposed that the latter can metabolize D-glucose under host-free conditions, as inferred from the production of ^14^CO_2_ from ^14^C-labeled D-glucose [74], [75], it was subsequently shown that the starting point for sugar catabolism in these bacteria is D-glucose-6-phosphate [65]. Moreover, experiments with substrates labeled at various carbon atoms revealed a combined action of glycolysis and PPP, yet complete TCA cycle activity could not be demonstrated [66], [74], [75]. In addition, anabolic reactions such as lipid and folate synthesis were detected in host-free *Chlamydiaceae* [76]–[79]. In contrast, while our findings from IRMS and the mass spectrometry-based metabolite analysis also indicate an involvement of the PPP in sugar metabolism and suggest occurrence of host-free anabolic reactions in *P. amoebophila* EBs, they additionally demonstrate that these bacteria are able to use non-phosphorylated D-glucose and that this sugar is at least to some extent completely catabolized via the TCA cycle (Fig. 3B, 5, and 6, Table 1).

Some of the host-free metabolic activities detected in *P. amoebophila* EBs are indeed not expected to occur in exactly the same manner in EBs of other chlamydial species due to known differences in their genomic repertoire. *P. amoebophila*, in contrast to *Chlamydiaceae*, encodes a glucokinase (*glk*, pc0935, UniProtKB Q6MCP0), which is required to activate D-glucose for metabolic reactions, and a complete enzyme set required for host-independent operation of the TCA cycle [14], [21]. Glucokinase, as well as most other enzymes involved in glycolysis/gluconeogenesis, PPP, TCA cycle, and in the electron transport chain, were recently also detected in the *P. amoebophila* EB proteome [40]. The mechanism by which D-glucose is imported in *P. amoebophila* remains to be elucidated, as the genome encodes a putative glucose-6-phosphate transporter (*uhpC*, pc0387, UniProtKB Q6ME88), the *C. pneumoniae* homolog of which has recently been functionally characterized [80], but does not provide evidence for a known importer for the non-phosphorylated sugar [14]. Early studies on host-free activities of *Chlamydiaceae* indicated that phosphorylation of D-glucose in suspensions of bacteria may also occur through the activity of a co-purified host-derived hexokinase [65]. However, this activity was shown to be strictly dependent on the availability of extensive amounts of ATP in the incubation medium [73]. Due to the pre-incubation step and the absence of externally added ATP we thus exclude significant contributions of host-derived kinase activity in the experiments presented in the present study.

Several lines of evidence suggest that host-free *P. amoebophila* EBs co-utilize medium-derived D-glucose with other internal or external carbon compounds that may even partially substitute for the sugar in its absence. Accordingly, during conditions of D-glucose deprivation, *i.e.* in DGM-L, CTC reducing activity was still detectable in a small proportion of the bacteria (Fig. 1 and S2F), suggesting that EBs may contain storage compounds, such as glycogen that may compensate D-glucose shortage for a period of time. The abundance patterns of differently labeled metabolic intermediates and the co-occurrence of unlabeled metabolites detected during the mass spectrometry-based analysis indicate an additional contribution of alternative substrates even when D-glucose is present in the extracellular medium. Thus, the fact that partially labeled citrate that contained predominately either three or four ^13^C atoms could be detected after incubation with fully labeled D-glucose (Fig. 6, Table 1) can only be explained by a mixed entry of labeled and unlabeled molecules into the TCA cycle. However, the high relative abundance of labeled metabolites in the PPP (59–94%, Fig. 6), as well as the fact that the absolute abundance of unlabeled molecules increased with proximity to the TCA cycle, contradicts extensive utilization of D-glucose from storage compounds during incubation in the nutrient-rich medium, but rather suggests a predominant co-utilization of substrates that can enter the central carbon metabolism at a level further downstream. These may include products from protein or lipid degradation or, even more likely considering their availability in the medium, imported amino acids. In fact, a potential utilization of amino acids is supported by a recent IRMS-based analysis conducted in our lab, which indicated that besides L-phenylalanine also L-glutamate, L-aspartate, and L-threonine could be imported by host-free *P. amoebophila* (data not shown). In addition, L-glutamate has also been proposed to represent a major carbon source for *C. trachomatis* based on their predicted metabolic repertoire [12]. However, the fact that CO_2_ production could not be detected for *P. amoebophila* incubated in DGM-L (Fig. 3B) and the rapid loss of its metabolic activity in this medium (Fig. 1 and S2), demonstrate that these additional carbon compounds are less effective than D-glucose, which thus appears to be an essential nutrient for maintenance of metabolic activity in host-free *P. amoebophila* EBs under the applied conditions. A similar substrate dependence may occur in *C. trachomatis* EBs, for which it was very recently shown that host-free metabolic activity can be greatly enhanced by the presence of D-glucose-6-phosphate [44].

The exclusive detection of only partially labeled six-carbon compounds in the glycolytic/gluconeogenic pathway, most likely derived from joining of ^12^C and ^13^C precursors, together with the observation that for all detected intermediates in the PPP a high proportion of the metabolite pool appeared to be fully labeled (Fig. 6), suggests that the latter represents the preferred route of D-glucose catabolism in host-free *P. amoebophila*. In addition to being an alternative pathway for the breakdown of sugars into products that can be further metabolized for ATP generation, the PPP represents the main route for regeneration of NADPH, a reducing agent that is not only required for lipid synthesis, but is also indispensable for maintaining the intracellular redox homeostasis, which in turn sustains protein function and counteracts oxidative stress [81]. An additional physiological function of D-glucose metabolism in EBs might be indicated by the detected synthesis of a disaccharide (Table 1) that could play a role in osmoprotection, though other functions, such as carbon storage, may be equally plausible.

The biological relevance of host-free activity of chlamydiae and its implications for their lifestyle have, to our knowledge, not been assessed before. A recent study that focused on *C. trachomatis* and several environmental chlamydiae reported differences in infectivity maintenance in nutrient-rich growth medium compared to sterile tap water [82]. The effects of the fundamentally different physicochemical properties of these incubation media, however, prohibited a correlation of the observed differences with nutrient availability. This is well illustrated by our observation of that *P. amoebophila* infectivity is markedly decreased after only a 2 h incubation in PBS or NaCl solution (Fig. 7A). In the present study, however, we demonstrate that the absence of D-glucose alone during host-free incubation of *P. amoebophila*, achieved by an exchange with its non-metabolizable stereoisomer, which does not affect the medium osmolarity, pH, or overall composition, is sufficient to cause a rapid decrease in the number of infectious particles (Fig. 7A–B). Likewise, exchange of D-glucose-6-phosphate with D-glucose resulted in a more rapid decline of *C. trachomatis* infectivity, consistent with the inability of this chlamydial species to utilize the non-phosphorylated compound (Fig. 7C–D). Addition of D-glucose to starved *P. amoebophila* failed to restore infectivity (Fig. S7), which indicates that a continuous supply of metabolizable substrates is required for the host-free survival of infectious EBs and thus demonstrates that metabolic activity in EBs is linked to their biological role as a dispersal stage. The more rapid decline of infectivity observed for *C. trachomatis* compared to *P. amoebophila* during host-free incubation may reflect differences in their metabolic potential, their requirement for host-free survival, or their adaptation to a host-free environment.

In conclusion, we provide evidence for D-glucose utilization and metabolic activity in host-free *P. amoebophila* EBs, which disagrees with the current perception of the infectious stage of chlamydiae as being metabolically inert and further establishes the chlamydial EB as being a developmental stage with a defined metabolic activity. The observed link between the availability of a metabolizable substrate and survival of infectious particles observed for *P. amoebophila* and *C. trachomatis* implies that the detection of metabolic activity in EBs is relevant for the main biological function of this infective stage, which thus appears to be more sensitive to its environment than has been thus far appreciated.

## Materials and Methods

### Cell Culture

*Acanthamoeba* sp. UWC1 containing *P. amoebophila* UWE25 [7] and symbiont-free isogenic amoebae were maintained at 20°C in TSY medium (30 g/l trypticase soy broth, 10 g/l yeast extract). HeLa 229 cells (ATCC, CCL-2.1) were cultivated at 37°C, 5% CO_2_ in Dulbecco's Modified Eagle's Medium (DMEM, Invitrogen) supplemented with 10% fetal bovine serum (PAA). *C. trachomatis* L2 was propagated in HeLa 229 cells by transfer of supernatant from infected to uninfected cultures every 2–3 days. Cultures were regularly screened for contamination by fluorescence microscopy using the DNA dye DAPI and fluorescence *in situ* hybridization (FISH). Mammalian cells were additionally shown to be free of contamination with *Mycoplasma* spp. by using the Venor GeM PCR kit (Minerva Biolab).The identity of the chlamydiae was verified by 16S rRNA gene sequence analysis, as described recently [83].

### Media for Extracellular Incubation

Extracellular incubations of *P. amoebophila* and *C. trachomatis* were conducted in media that were based on the chemically defined *Acanthamoeba* medium DGM-21A [42], but that, as a modification, contained L-phenylalanine (5.4 mM) instead of the racemic mixture DL-phenylalanine and were additionally supplemented with 0.25 g/l NaHCO_3_. Specific media used in this study included DGM-D, which like the original DGM-21A contained 83.2 mM D-glucose, DGM-D/2 with a reduced D-glucose concentration of 41.6 mM, DGM-L containing 83.2 mM L-glucose instead of D-glucose, DGM-DL containing both 83.2 mM D-glucose and 83.2 mM L-glucose, DGM-D6P containing 83.2 mM D-glucose-6-phosphate instead of D-glucose, and DGM-DD6P containing both 83.2 mM D-glucose and 83.2 mM D-glucose-6-phosphate. In addition, DGM-D-based media in which certain endogenous substrates were completely replaced by stable isotope-labeled variants were also used. These media included DGM-D-13C (containing fully ^13^C-labeled D-glucose (D-[U-13C6]-glucose, 99%)), DGM-D-1-13C (containing D-glucose labeled at carbon 1 (D-[1-13C]-glucose, 98–99%)), DGM-D-6-13C (containing D-glucose labeled at carbon 6 (D-[6-13C]-glucose, 99%)), and DGM-D-13C15N (containing D-[U-13C6]-glucose and fully ^13^C^15^N-labeled L-phenylalanine (L-[U-13C9,15N]-phenylalanine, 97–99%)). The composition of all incubation media is summarized in Table S2. Stable isotope-labeled substrates were purchased from Euriso-top.

### Purification of *P. amoebophila* EBs and RBs

Recently, we developed a protocol for an efficient purification of *P. amoebophila* EBs and RBs and investigated the purity of obtained fractions by an ultrastructural analysis (Fig. S1) [43]. In the current study a slightly modified protocol, including thicker layers in the density gradients for improved phase separation, was applied. Briefly, after host cell disruption and filtration, released bacteria were directly subjected to density gradient centrifugation using a gradient consisting of 3.5 ml 30% (v/v) gastrografin (Bayer Schering Pharma) in Page's amoebic saline (PAS; 0.12 g/l NaCl, 0.004 g/l MgSO_4_×7H_2_O, 0.004 g/l CaCl_2_×2H_2_O, 0.142 g/l Na_2_HPO_4_, 0.136 g/l KH_2_PO_4_) and 3.5 ml 50% (w/v) sucrose. The gradient applied during the second centrifugation consisted either of 3.5 ml 34% (v/v) and 3.5 ml 40% (v/v) gastrografin or of 3.5 ml 40% (v/v) and 3.5 ml 46% (v/v) gastrografin. RB-enriched and intermediate fractions were collected at the 34/40% or 40/46% interface, respectively. A highly enriched EB fraction was collected at the bottom of the 40/46% gradient. For applications requiring large amounts of biomass, such as the analysis of biomass by IRMS or the preparation of metabolite extracts for ICR/FT-MS and UPLC-MS analysis, the EB-enriched pellet of the 34/40% gastrografin gradient (in this study explicitly referred to as “EB-enriched fraction”) was used instead. Purified bacteria were washed once in 10 ml PAS (12 860× g, 10 min), resuspended in DGM-D, and analyzed immediately or after a 40 h pre-incubation (27°C, 200 rpm). Bacteria that were heat-inactivated (30 min, 80°C, 700 rpm) directly before assessment of activity were included as control when indicated.

### Preparation of Host Cell Lysates

Host cell lysates, that were included as negative control when indicated, were prepared analogously to the first steps applied during the purification of bacteria [43]. Briefly, uninfected *Acanthamoeba* sp. UWC1 were harvested at 3200× g, washed with PAS, and resuspended in 6.5 ml sucrose-phosphate-glutamate buffer (75 g/l sucrose, 0.52 g/l KH_2_PO_4_, 1.53 g/l Na_2_HPO_4_×2H_2_O, 0.75 g/l glutamic acid) per 1 g wet weight. After cell disruption on ice by using a dounce homogenizer (Wheaton), the suspension was filtrated (1.2 µm). Cell debris was collected at 12 860× g (10 min, 4°C), resuspended in DGM-D, and analyzed immediately or after a 40 h pre-incubation (27°C, 200 rpm).

### Fluorescence Microscopic Detection of Respiratory Activity

Respiratory activity in fractions of living or heat-inactivated bacteria and in host lysates was analyzed immediately or after pre-incubation. For that purpose, bacteria or cell debris were collected by centrifugation (10 620× g, 10 min, 4°C), resuspended in 220 µl DGM-D containing 5 mM CTC (Sigma-Aldrich), and incubated for 2 h (27°C, 200 rpm). For the assessment of the effect of D-glucose deprivation, purified bacteria were washed twice in 1 ml DGM-L directly after the purification and both the pre-incubation and the incubation with CTC were subsequently conducted in DGM-L. After the incubation with CTC, bacteria and amoebal cell debris were collected at 10 620× g (10 min), fixed in 400 µl 4% formaldehyde (15 min, room temperature), and washed once with 1 ml PBS (10 mM Na_x_PO_4_, 0.76% NaCl (if not stated otherwise), pH 7.3). Pellets were then resuspended in a small volume PBS and transferred to microscope slides. Bacteria and cell debris were dried (46°C), stained with DAPI (0.5 µg/ml in PBS, 10 min), washed once with PBS, and embedded in mowiol [84]. Images were taken with a CCD camera (AxioCam HRc; Carl Zeiss) connected to an epifluorescence microscope (Axioplan 2 imaging; Carl Zeiss). The percentage of DAPI-stained bacteria containing red fluorescent CTC crystals was determined. At least 1500 bacteria per sample, 500 for each of three replicate wells on the microscope slide, were counted. In addition, the efficiency of DAPI staining, *i.e.* the percentage of bacteria observable in DIC that could be detected by DAPI staining, was also assessed. For that purpose, at least 450 bacteria per sample, including 150 per replicate well, were considered. Data were collected for three independent experiments (throughout this study, the term “independent experiments” refers to experiments conducted with separate purifications of bacteria).

### Fluorescence Microscopic Detection of D-Glucose Uptake

Purified living and heat-inactivated EBs were analyzed for D-glucose uptake either immediately or after pre-incubation. For that purpose, bacteria were collected by centrifugation (10 620× g, 10 min, 4°C), resuspended in 220 µl DGM-D/2 containing 100 µM 2-NBDG (Invitrogen), and incubated for 10 h (27°C, 200 rpm). After the incubation, bacteria were washed once with 1 ml PBS and transferred to microscope slides. Images were obtained as described above. The percentage of stained cells was determined for three separate incubations (representing 2 independent experiments), for each of which at least 500 bacterial cells were evaluated.

### Sample Preparation for IRMS Analysis

For the detection of ^13^C-D-glucose uptake and catabolism by IRMS, a pre-incubated EB-enriched fraction or a highly pure EB fraction were applied for the first and second experimental approach, respectively. After pre-incubation, the suspension of bacteria was mixed by vortexing and transferred to 1.5 ml tubes, so that each tube contained the same number of cells. Bacteria were then collected by centrifugation (10 620× g, 10 min), resuspended in 1 ml of the respective incubation medium (DGM-D, DGM-L, DGM-D-13C, DGM-D-1-13C, or DGM-D-6-13C), and transferred to 15 ml glass vials that were subsequently closed with gas impermeable butyl rubber stoppers (GMT) and incubated for 48 h (27°C, 200 rpm). Prior to the incubation in DGM-L, bacteria were washed twice in 1 ml of this medium. When indicated, incubations of heat-inactivated bacteria or host cell lysates in DGM-D-13C were included as control. After the 48 h period, gas and biomass samples were collected and pre-processed for IRMS analysis. For the CO_2_ measurements, defined volumes (between 6.5 and 7.5 ml per sample) of the head-space of the incubations, as well as of parallel incubations of bacteria-free media (that served as blanks for the gas measurements), were collected with a syringe, transferred to evacuated glass tubes (exetainers, 12 ml, Labco), and brought to a volume of 15 ml with N_2_ gas prior to IRMS analysis. For the biomass measurements, incubated bacteria were collected by centrifugation (20 820× g, 5 min, 4°C) and washed twice with 1 ml PBS. Prior to the wash steps, bacteria incubated in DGM-D, which served as blank for biomass measurements, were shortly washed in DGM-D-13C to allow substrate adsorption to the surface of the bacteria. Bacterial pellets were finally heat-inactivated (80°C, 10 min), dried for 6 h in a speedvac (Concentrator 5301, Eppendorf) and subsequently overnight at 60°C. Defined amounts of biomass (between 0.3 and 0.5 mg per sample) were transferred to tin capsules and subjected to IRMS analysis. Prior to the processing of biomass for IRMS, aliquots were withdrawn from bacterial suspensions for the quantification of bacterial particles using a previously described procedure [83]. Applied bacterial numbers were similar between replicate experiments (between 3.9×10^8^ and 5.9×10^9^ or between 6.3×10^8^ and 1.0×10^9^ bacteria per incubation, for the first and second experimental approach, respectively). The applied amount of host cell lysate exceeded that of the bacterial biomass, based on the size of the pellets. Samples for IRMS measurements were obtained from three independent experiments each consisting of two replicate incubations per condition. An exception was the incubation in DGM-L, for which only two independent experiments were conducted.

### IRMS Measurements and Data Analysis

Analysis of bacterial biomass for total carbon and C isotopes (^13^C, ^12^C) was conducted using an elemental analyzer (EA 1110, CE Instruments) interfaced via a ConFlo III device (Thermo Finnigan) to a continuous flow stable isotope ratio mass spectrometer (DeltaPLUS, Thermo Finnigan). A mixture of proline and sucrose was used as standard, which was regularly calibrated against international standards (IAEA) for ^13^C and against atropine for total C content. Stable isotopes (^13^C, ^12^C) in CO_2_ of gas samples were analyzed against CO_2_ reference gas by an isotope ratio mass spectrometer (Delta Advantage V, Thermo Fisher Scientific) coupled to a headspace gas sampler (GasBench II, Thermo Fisher Scientific) with a GC-PAL autosampler (CTC Analytics). CO_2_ reference gas was calibrated using ISO-TOP gas standards with certified ^13^C concentrations (Air Liquide) and counterchecked by elemental analysis coupled with IRMS of international standards (IAEA). The averages of peak area and At%^13^C of three consecutive injections of each sample were used to calculate concentrations of CO_2_ and ^13^C, respectively. For calibration of CO_2_ concentration, gas standards containing 495 and 1020 ppm were analyzed together with the samples. For gas samples, measures for bacterial CO_2_ production and ^13^C enrichment in CO_2_ were calculated based on equations (1) and (2), respectively.
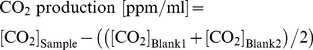
(1)

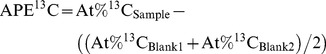
(2)


In these equations, [CO_2_] and At%^13^C represent the measured values for CO_2_ (in ppm/ml) and At%^13^C for the sample and for the two corresponding blank incubations (*i.e.* incubations of bacteria-free media) of the respective experiment. Based on the APE^13^C observed for incubations in media with differently labeled D-glucose isotopologs the contributions of different metabolic pathways to ^13^CO_2_ release were estimated using a script written in Python 2.7.2. The working procedure of the calculation is depicted in Fig. S4. Bacterial ^13^C incorporation (per mg DW) was calculated for each sample by applying equation (3).
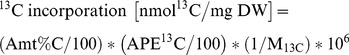
(3)


In this equation, Amt%C indicates the measured proportional amount of carbon in the sample and M_13C_ the atomic mass of ^13^C (13.00335). The APE^13^C is calculated from the measured At%^13^C of the sample by subtracting the mean of the At%^13^C of the two blank incubations of the respective experiment (*i.e.* bacteria incubated in DGM-D) (as described in equation (2)).

### Sample Preparation for Metabolite Analysis

For the analysis of metabolite extracts, a pre-incubated EB-enriched fraction was mixed by vortexing and transferred to 1.5 ml tubes, so that each tube contained the same number of cells. After centrifugation (10 620× g, 10 min), bacteria were resuspended in 1 ml of the respective incubation medium (DGM-D or DGM-D-13C15N) and incubated for 48 h (27°C, 200 rpm). Bacteria that were heat-inactivated prior to incubation in DGM-D-13C15N were included as control. For preparation of metabolite extracts, bacteria were centrifuged (20 820× g, 5 min, 4°C) and washed once with 1.5 ml cold PBS. Bacterial pellets were then resuspended in 400 µl cold (−20°C) methanol (≥99.9%, CHROMASOLV, Fluka), frozen in liquid nitrogen (1 min), and thawed on ice, followed by two cycles consisting of vortexing (30 sec) and 5 min incubation on ice. After centrifugation (20 820× g, 5 min, 4°C) extracts were transferred to pre-cooled 1.5 ml tubes and pellets were extracted a second time with 400 µl of a cold (−20°C) 1∶1 mixture of methanol (≥99.9%, CHROMASOLV) and water (LC-MS Ultra CHROMASOLV, Fluka) by the same procedure as described above. Both extracts were pooled and stored at −80°C until analysis. As extraction blank, empty 1.5 ml tubes were subjected to the same extraction procedure. Metabolite extracts were obtained for three independent experiments each consisting of two replicate incubations per incubation condition. Bacterial numbers, which were assessed by quantification of bacterial particles in an aliquot that was withdrawn from the bacterial suspension during the pre-incubation, were highly similar between replicate experiments, ranging from 2.6×10^9^ to 2.8×10^9^ bacteria per incubation.

### ICR/FT-MS Measurements and Data Analysis

Ultrahigh resolution mass spectra were acquired on a solariX ICR/FT mass spectrometer (Bruker Daltonics) equipped with an Apollo II electrospray source (Bruker Daltonics) and a 12 Tesla super conducting magnet (Magnex Scientific). The mass spectrometer was tuned in order to obtain highest sensitivity for metabolites in the m/z range of about 150 to 500 Da in broad band detection mode with a time domain transient of 2 Megaword. The instrument was calibrated with a 1 ppm arginine solution. A mass error below 100 ppb and a resolving power of ∼300 000 at m/z 300 was achieved. Negative electrospray ionization (ESI) mode was chosen due to favored ionization of carbohydrates and their metabolic derivatives by proton loss or chloride attachment [85]. Diluted extracts (1∶100 in methanol (≥99.9%, CHROMASOLV) were cooled (8°C) and injected (2 µl/min flow rate) through a Gilson autosampler (sample changer 223, Gilson). In total 600 scans were acquired for one spectrum of each sample. The obtained spectra were internally calibrated against naturally abundant fatty acids and analyzed in DataAnalysis 4.0 SP2 (Bruker Daltonics). Mass lists were generated with a signal-to-noise ratio (S/N) of four, exported, and combined to one data matrix by applying a 1 ppm window [86]. Subsequently, mass lists were filtered very conservatively. Masses that were also detected in the extraction blanks were excluded if their detected intensity in the samples did not exceed ten times the detected intensity in the blank. Furthermore, for each incubation condition, masses found in less than two out of the three independent biological experiments were also excluded. Detected metabolites in extracts from DGM-D-incubated EBs were annotated with MassTRIX (<1 ppm) [61]. For the detection of ^13^C-labeled metabolites an application of mass difference-based networks [87] was developed and applied. Hereby, mass differences of peaks detected in samples from DGM-D- and DGM-D-13C15N-incubated bacteria were compared by a polynomial-time algorithm. Labeled metabolites were considered to be present in extracts of DGM-D-13C15N-incubated EBs when peaks corresponding to the exact mass of the unlabeled metabolites were detected in the DGM-D-incubated controls and observed mass shifts were consistent with the exchange of ^12^C by ^13^C atoms.

### UPLC-MS Measurements and Data Analysis

UPLC-MS analysis of metabolite extracts was conducted on an UHR QqToF instrument (maXis, Bruker Daltonics) hyphenated to an ACQUITY UPLC (Waters). Prior to injection, extracts were dried (SpeedVac Concentrator, Savant SPD 121P, Thermo Fisher Scientific) and re-solved in solvent A (see below), using half of the original sample volume. Separation was performed on a ACQUITY UPLC BEH Amide column (150×2.1 mm, 1.5 µm, Waters) using a 3 min gradient from 10% to 90% solvent B, followed by 2 min plateau on 90% B (solvent A: 80% acetonitrile, 20% water, 0.1% (v/v) ammonium hydroxide; solvent B: 70% water, 30% acetonitrile, 0.1% (v/v) ammonium hydroxide). A column equilibration time of 5 min was applied after each analysis. The flow rate was optimized to 0.1 ml/min with a column temperature of 45°C. ToF mass spectra were acquired in negative ESI mode. Parameters were tuned for best resolution and sensitivity in the mass range of about 100 to 400 Da. A quality control consisting of an aliquot of all samples and a mixture of different standard compounds (including raffinose, ribose, arabinose, galactose, fructose, fucose, gentiobiose, erythrol, glucose, pyruvate, and citrate) was used to monitor drifts in retention time and mass accuracy. The acquired spectra were calibrated internally against naturally abundant fatty acids (DataAnalysis 4.0 SP2, Bruker Daltonics) and exported to MZmine 2.7 [88] for data evaluation. Annotation of the m/z features in spectra from DGM-D-incubated bacteria was carried out with MassTRIX applying a maximal mass error of 0.005 Da [61]. Corresponding masses of fully or partially labeled metabolites were calculated and their presence in spectra of DGM-D-13C15N-incubated samples was checked manually. Masses of putatively labeled metabolites were taken in consideration if the retention time and peak shape matched with the corresponding parameters of unlabeled metabolites detected in DGM-D-incubated EBs and the mass difference between putatively labeled and unlabeled metabolites was consistent with a shift corresponding to the exchange of ^12^C by ^13^C atoms.

### Infectivity Assay (*P. amoebophila*)

For the analysis of the effect of nutrient availability on infectivity, *P. amoebophila* were purified from host cells using a protocol without density gradient centrifugation in order to mimic more closely the natural situation of bacterial dispersal in the environment. Briefly, *Acanthamoeba* sp. UWC1 infected with *P. amoebophila*, as well as released bacteria in supernatants of respective cultures, were harvested and washed, followed by disruption of host cells, as described for the purification of EBs by density gradient centrifugation [43]. After filtration (1.2 µm), bacteria were collected by centrifugation (12 800× g, 10 min), washed once with 20 ml PBS, and then resuspended in a small volume of PBS. A small aliquot was withdrawn for the quantification of bacterial particles (see above). The bacterial suspension was then transferred into 1.5 ml tubes for parallel host-free incubations in different media, including a 0.6% NaCl solution, PBS (containing 0.6% NaCl), DGM-D, DGM-L, and DGM-DL. After centrifugation, bacteria were resuspended in the respective media and incubated at 27°C (200 rpm) for 2, 48, 94, or 168 h before being added to amoebae that had been seeded into a 24-well plate (Nunc; 1×10^5^ cells per well) at a multiplicity of infection (MOI) of 5. Amoebae and bacteria in each well were mixed by pipetting, followed by 15 min incubation at 27°C, centrifugation (130× g, 15 min, 23°C), and incubation at 27°C for 48 h. Amoebae were then transferred to microscope slides, fixed with 4% formaldehyde (10 min, room temperature), and washed with PAS. For the detection of bacteria by FISH, cells were dehydrated by incubation in increasing concentrations of ethanol (50%, 80% and 96%, 3 min incubation with each) and hybridized with a combination of two Cy3-labeled probes, targeting different positions at the 16S rRNA, to increase signal strength. Applied probes (purchased from Thermo Fisher Scientific) included the *Chlamydiae*-specific probe Chls-0523 [89] and the probe E25-454 (5′-GGA TGT TAG CCA GCT CAT-3′) that had been designed to target *P. amoebophila*. Hybridization occurred at 46°C for 1.5 h at a formamide concentration of 20%, using hybridization and wash buffers described elsewhere [90]. Cells were embedded in mowiol and images were taken as described above. The percentage of infected cells (containing at least six intracellular bacteria) was determined taking into account a minimum of 600 cells per sample. Infectivity was expressed relative to the infectivity observed for bacteria incubated for 2 h in DGM-D. Data were collected for at least three independent experiments, each consisting of three parallel host-free incubations per incubation medium and duration.

### Infectivity Assay (*C. trachomatis*)

HeLa 229 cells infected with *C. trachomatis* were harvested at 40 h p.i (when significant host cell lysis was first observed), washed with DPBS (Invitrogen), resuspended in SPG buffer [83], and disrupted by two rounds of freezing (dry ice/ethanol bath) and thawing (37°C). After removal of host cell debris by centrifugation (250× g, 5 min, 4°C), the supernatant was filtered (1.2 µm). Bacteria were collected by centrifugation (15 557× g, 10 min, 4°C) and resuspended in SPG buffer. The bacterial suspension was then transferred into 1.5 ml tubes for parallel host-free incubations in different media, including DPBS, DGM-D, DGM-L, DGM-D6P, and DGM-DD6P. After centrifugation (12 851× g, 10 min, 4°C), bacteria were resuspended in the respective media and incubated at 37°C (200 rpm) for 30 min, 2 h, 6 h, or 24 h before being added to HeLa 229 cells that had been seeded into a 24-well plate (Nunc; 7×10^4^ cells per well). Multi-well plates were incubated at 37°C, 5% CO_2_ for 24 h. Cells were fixed with 4% formaldehyde (1 h, room temperature) and washed with DPBS. For the detection of bacteria by immunostaining, cells were first permeabilized in 0.2% Triton-X-100 in PBS for 15 min, followed by incubation in blocking solution (2% BSA in PBS, 20 min), and subsequent incubations with primary antibodies (raised against recombinant *P. amoebophila* heat shock protein DnaK [91]) and secondary antibodies (Cy3-labled, Dianova) diluted in blocking solution (for 1 h each). HeLa 229 cells were counter-stained with HCS CellMask Deep Red cytoplasmic stain (Life technologies; 10 µg/ml, 20 min) and DNA was stained with DAPI (0.5 µg/ml in PBS, 10 min). Cells were embedded in Mowiol and images were taken at a confocal laser scanning microscope (TCS SP8 X, Leica). The percentage of infected cells was determined taking into account at least 300 cells per sample. Infectivity was expressed relative to the infectivity observed for bacteria incubated for 30 min in DGM-D6P. Data were collected for three parallel host-free incubations per incubation medium and duration.

### Statistical Analysis

Throughout the study values are given as means with standard deviations. Unpaired two-sided student's t-test and one-way ANOVA were carried out using the software PASW statistics 17.0 (SPSS Inc.). Dunnett's T3 test was chosen as post-hoc test for ANOVA due to the fact that experimental data did frequently not comply with the criteria of variance homogeneity. The following notation of significance levels was used throughout the study: ***, p≤0.001; **, p≤0.01; *, p≤0.05. The multivariate modeling was done in SIMCA-P 9 (Umetrics). PCA models were used for data visualization and discovery of naturally occurring differences in the metabolite pattern of living and heat-inactivated EBs and of EBs incubated in different media. Mean centering in combination with unit variance scaling was applied. Data were further analyzed with PLS-DA to extract the most discriminative compounds characterizing living EBs compared to heat-inactivated bacteria. A seven-fold cross-validation, as well as a permutation test using 200 iterations, was conducted for model validation.

## Supporting Information

Figure S1 **Transmission electron micrographs of purified** ***P. amoebophila*** **developmental stages.** *P. amoebophila* developmental forms were purified from amoebal host cells and separated from each other by density gradient centrifugation, using a previously established protocol [43]. TEM was carried out as described in the respective study [43]. Micrographs of a highly pure EB fraction (collected below 46% gastrografin), as well as of an RB-enriched and an intermediate fraction (collected above 40% gastrografin or at the 40/46% interface, respectively), are shown. A quantitative evaluation indicated a high enrichment of the replicative stage in RB fractions (5% EB, 34% IB, 61% RB), whereas EB fractions were highly enriched in EBs (76% EB, 16% IB, 8% RB) and intermediate fractions represented a more uniform mixture of all stages (38% EB, 35% IB, 27% RB). Bacteria were classified as RBs, EBs, and IBs based on their characteristic morphological features. Thus, bacteria containing reticulated material and a relaxed nucleoid were considered as mature RBs (white arrows), bacteria containing only electron-dense and electron-lucent material were considered as mature EBs (black arrows), and intermediate morphologies were considered as IBs (gray arrowheads). The bar indicates 1 µm.
(TIF)

Figure S2 **Visualization of respiratory activity in** ***P. amoebophila*** **developmental stages and the effect of D-glucose deprivation.** Activity of *P. amoebophila* developmental forms was assessed by application of CTC as indicator for respiration. RB (A), intermediate (B), and EB (C) fractions of *P. amoebophila* were subjected to host-free incubation in DGM-D containing 5 mM CTC either immediately after purification or after a 40 h pre-incubation in DGM-D. A heat-inactivated EB fraction (D) and a lysate of uninfected amoebae (E) were included as controls. The effect of D-glucose deprivation on EB activity was tested by replacement of DGM-D with DGM-L during pre-incubation and incubation with CTC (F). Incubation with CTC was followed by formaldehyde fixation and DNA staining with DAPI. Fluorescence and corresponding DIC images are shown (reduced CTC, red; DAPI, blue). The bar indicates 10 µm.
(TIF)

Figure S3 **D-glucose uptake in host-free** ***P. amoebophila*** **EBs revealed by IRMS.** An EB-enriched fraction of *P. amoebophila* was pre-incubated for 40 h in DGM-D, followed by 48 h incubation in DGM-D or DGM-D-13C and subsequent analysis of bacterial biomass by IRMS. Heat-inactivated bacteria incubated in DGM-D-13C were included as control. The amount of incorporated ^13^C (in nmol ^13^C/mg DW) in DGM-D-13C-incubated bacteria was calculated by considering the ^13^C content of DGM-D-incubated bacteria as blank. Diamonds indicate results from individual replicates, bars display mean values. Results from three independent experiments each consisting of two replicate incubations per condition are shown. Bacterial numbers applied per incubation were similar between replicate experiments (between 3.9×10^9^ and 5.9×10^9^ bacteria). The observed difference in ^13^C incorporation between living and heat-inactivated bacteria was statistically significant (t-test; ***, p≤0.001).
(TIF)

Figure S4 **Working scheme for the estimation of the contributions of different metabolic pathways to ^13^CO_2_ release.** In respect to the release of certain carbon atoms from D-glucose as CO_2_, three major metabolic scenarios (A–C) were distinguished, which are indicated in the gray box and will here be termed “pathways” for simplicity. The pathway ratio was calculated based on the APE^13^C in CO_2_ observed for incubations in media with different labeled D-glucose isotopologs (blue box). Initially, these experimental values were used to calculate an experimental APE^13^C ratio and the pathway ratio was set to 0∶0∶1 (A∶B∶C), assuming that all D-glucose is completely catabolized (red box). Based on this pathway ratio a corresponding expected ratio of APE^13^C for the differently labeled substrates was calculated, assuming that pathways A, B, and C lead to a release of 1.83, 1, and 6, respectively, carbons from D-[U-13C6]-glucose, 0, 1, and 1, respectively, carbons from D-[1-13C]-glucose, and 0, 0, and 1, respectively, carbons from D-[6-13C]-glucose. The assumed release of 1.83 carbon atoms per molecule D-[U-13C6]-glucose by pathway A was based on the assumption that pyruvate, as starting molecule for scenario A, is produced with equal probability from glycolysis and PPP activity, the latter of which already releases carbon 1 as CO_2_ and thus yields a lower amount of pyruvate per molecule glucose. The calculated expected APE^13^C ratio was then compared to the experimental APE^13^C ratio. This was followed by stepwise adjustments of B and A until the expected APE^13^C ratio coincided with the experimentally observed ratio (yellow box). The finally obtained pathway ratio represents an estimation of the contributions of the three considered metabolic scenarios to ^13^CO_2_ release by host-free *P. amoebophila* EBs (green box).
(TIF)

Figure S5 **Comparison of ICR/FT-MS metabolite profiles from DGM-D- and DGM-D-13C15N-incubated living and heat-inactivated EBs.** In (A) representative ESI(-)ICR/FT-MS spectra of DGM-D- and DGM-D-13C15N-incubated living bacteria, as well as of DGM-D-13C15N-incubated inactivated bacteria, are shown. Spectra illustrate the m/z range of 150–800. In addition, an enlarged view on the m/z range of 175–190 (indicated in gray in the overview spectra), is displayed. A comparison of spectra from living bacteria incubated in the two different media revealed a shift of the D-glucose peak from m/z ∼179 (^12^C-D-glucose) to m/z ∼185 (^13^C-D-glucose), while the remaining pattern was unaltered. The exchange of D-glucose and L-phenylalanine in the incubation medium against their stable isotope-labeled analogs thus did not profoundly change the metabolite profile of *P. amoebophila* EBs. This visual impression, as well as the effect of heat-inactivation, was further tested by pairwise PCA comparisons of spectra from DGM-D- and DGM-D-13C15N-incubated living bacteria (B), and of spectra from DGM-D-1315N-incubated inactivated bacteria with those from DGM-D-incubated living EBs (C) or DGM-D-13C15N-incubated living EBs (D). Note that this analysis revealed a separation of spectra from living and dead bacteria, whereas no separation could be observed for samples from living bacteria incubated in the two different media.
(TIF)

Figure S6 **PLS-DA analysis for the extraction of metabolites discriminative for living compared to heat-inactivated EBs.** ICR/FT-MS spectra were analyzed by PLS-DA to extract the most discriminative compounds characterizing living compared to inactivated EBs. The PLS-DA model included data from DGM-D-incubated living and DGM-D-13C15N-incubated inactivated EBs. The assignment of discriminative and non-discriminative metabolites to KEGG pathways is displayed in Fig. 4.
(TIF)

Figure S7 **Lack of infectivity restoration by addition of D-glucose to starved** ***P. amoebophila*** **.** *P. amoebophila* were harvested from amoebal host cell cultures followed by host-free incubation for the indicated periods of time in DGM-D or DGM-L. Subsequently, bacteria incubated in DGM-L were supplemented with D-glucose (83.2 mM final concentration). After an additional incubation of the bacteria for 15 min (27°C, 200 rpm), amoebae were infected at a MOI of 4.3 and the percentage of infected cells was assessed at 48 h p.i. after detection of intracellular bacteria by FISH. The observed infectivity, relative to that observed for 2 h incubation in DGM-D, is shown. Data represent means and standard deviations of three independent experiments, each consisting of three replicate host-free incubations. For each sample at least 600 cells were counted. Statistically significant differences in infectivity observed between the incubation media at selected time points after start of host-free incubation are indicated (t-test; ***, p≤0.001; **, p≤0.01).
(TIF)

Table S1 **Non-annotated ^13^C-labeled metabolites detected by ICR/FT-MS in DGM-D-13C15N-incubated EBs.**
(DOCX)

Table S2 **Media for extracellular incubation of** ***P. amoebophila*** **.**
(DOCX)
