# Supplementary figures and images for: Metabolic Features of Protochlamydia amoebophila Elementary Bodies – A Link between Activity and Infectivity in Chlamydiae
Source: PLoS Pathog. 2013 Aug 8;9(8):e1003553. doi: 10.1371/journal.ppat.1003553 (PMC3738481; doi:10.1371/journal.ppat.1003553)

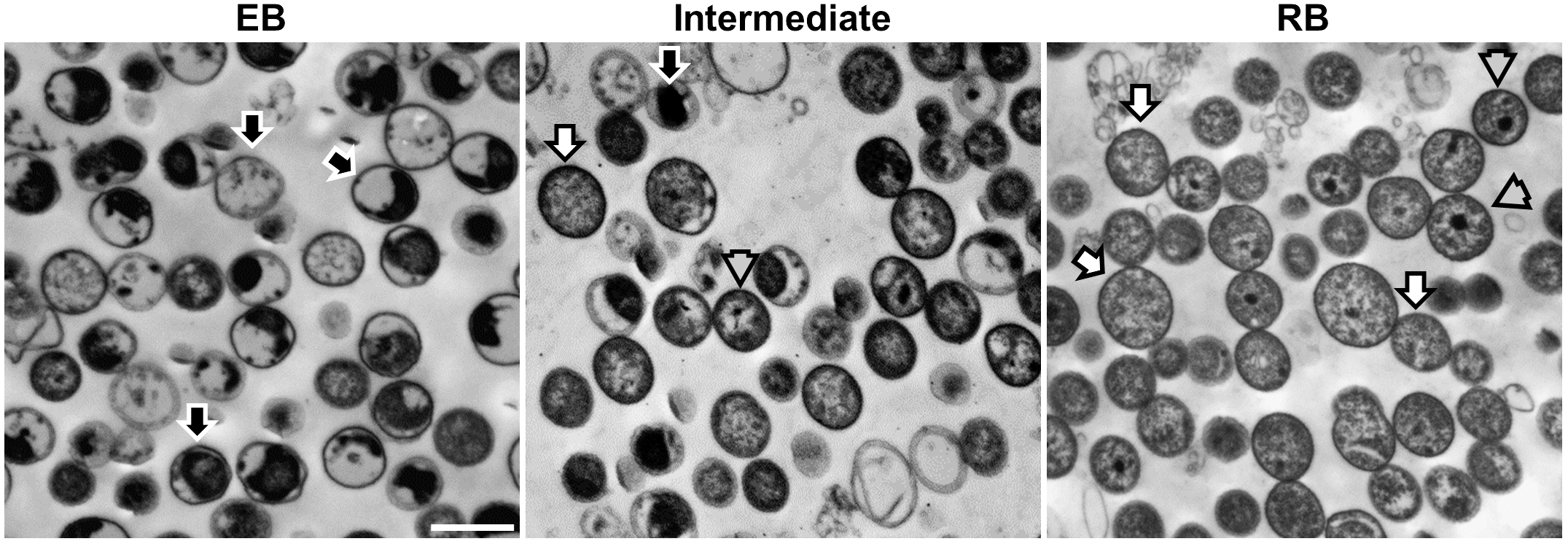

Supplement: Figure S1 — Transmission electron micrographs of purified P. amoebophila developmental stages. P. amoebophila developmental forms were purified from amoebal host cells and separated from each other by density gradient centrifugation, using a previously established protocol [43]. TEM was carried out as described in the respective study [43]. Micrographs of a highly pure EB fraction (collected below 46% gastrografin), as well as of an RB-enriched and an intermediate fraction (collected above 40% gastrografin or at the 40/46% interface, respectively), are shown. A quantitative evaluation indicated a high enrichment of the replicative stage in RB fractions (5% EB, 34% IB, 61% RB), whereas EB fractions were highly enriched in EBs (76% EB, 16% IB, 8% RB) and intermediate fractions represented a more uniform mixture of all stages (38% EB, 35% IB, 27% RB). Bacteria were classified as RBs, EBs, and IBs based on their characteristic morphological features. Thus, bacteria containing reticulated material and a relaxed nucleoid were considered as mature RBs (white arrows), bacteria containing only electron-dense and electron-lucent material were considered as mature EBs (black arrows), and intermediate morphologies were considered as IBs (gray arrowheads). The bar indicates 1 µm. (TIF) [file ppat.1003553.s001.tif]

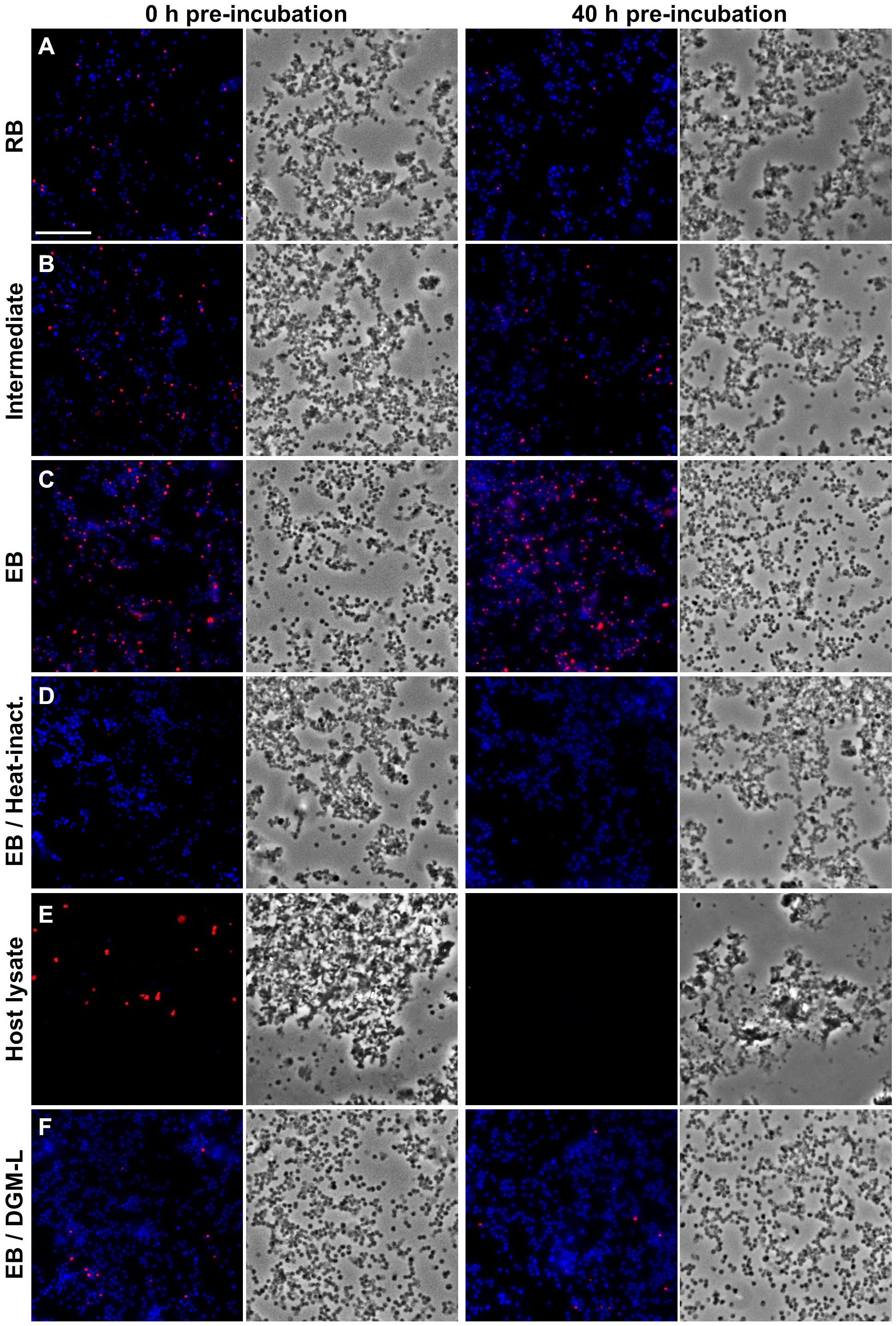

Supplement: Figure S2 — Visualization of respiratory activity in P. amoebophila developmental stages and the effect of D-glucose deprivation. Activity of P. amoebophila developmental forms was assessed by application of CTC as indicator for respiration. RB (A), intermediate (B), and EB (C) fractions of P. amoebophila were subjected to host-free incubation in DGM-D containing 5 mM CTC either immediately after purification or after a 40 h pre-incubation in DGM-D. A heat-inactivated EB fraction (D) and a lysate of uninfected amoebae (E) were included as controls. The effect of D-glucose deprivation on EB activity was tested by replacement of DGM-D with DGM-L during pre-incubation and incubation with CTC (F). Incubation with CTC was followed by formaldehyde fixation and DNA staining with DAPI. Fluorescence and corresponding DIC images are shown (reduced CTC, red; DAPI, blue). The bar indicates 10 µm. (TIF) [file ppat.1003553.s002.tif]

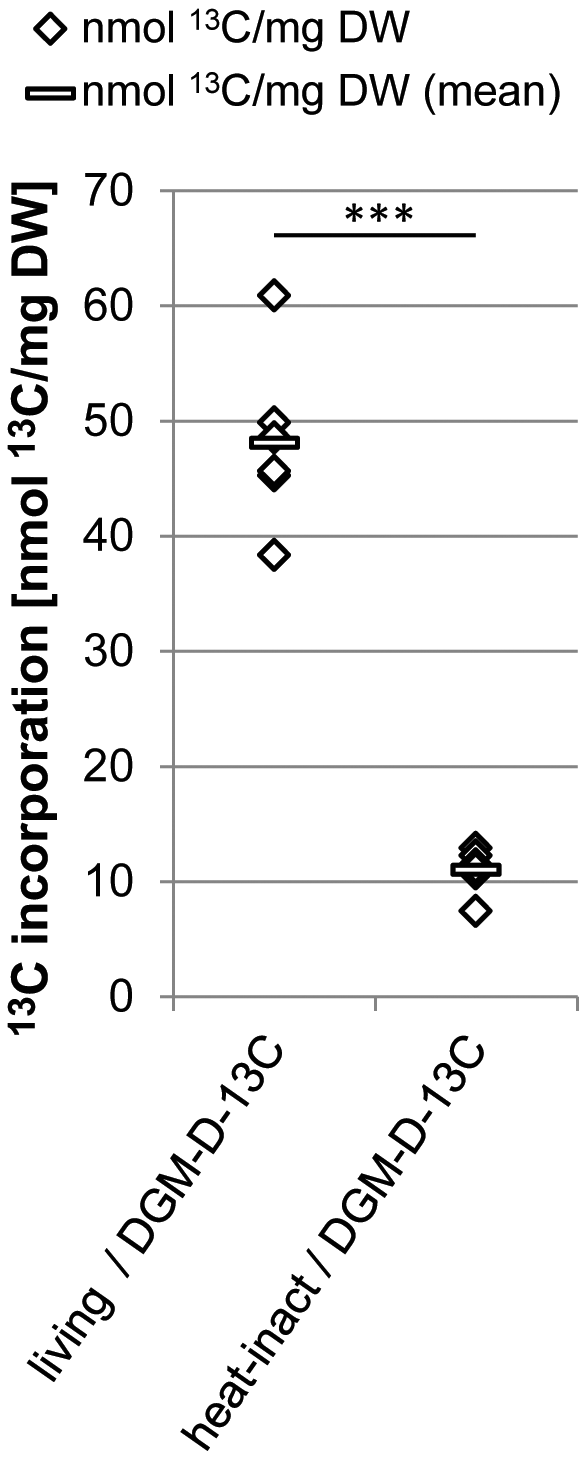

Supplement: Figure S3 — D-glucose uptake in host-free P. amoebophila EBs revealed by IRMS. An EB-enriched fraction of P. amoebophila was pre-incubated for 40 h in DGM-D, followed by 48 h incubation in DGM-D or DGM-D-13C and subsequent analysis of bacterial biomass by IRMS. Heat-inactivated bacteria incubated in DGM-D-13C were included as control. The amount of incorporated 13C (in nmol 13C/mg DW) in DGM-D-13C-incubated bacteria was calculated by considering the 13C content of DGM-D-incubated bacteria as blank. Diamonds indicate results from individual replicates, bars display mean values. Results from three independent experiments each consisting of two replicate incubations per condition are shown. Bacterial numbers applied per incubation were similar between replicate experiments (between 3.9×10^9^ and 5.9×10^9^ bacteria). The observed difference in 13C incorporation between living and heat-inactivated bacteria was statistically significant (t-test; ***, p≤0.001). (TIF) [file ppat.1003553.s003.tif]

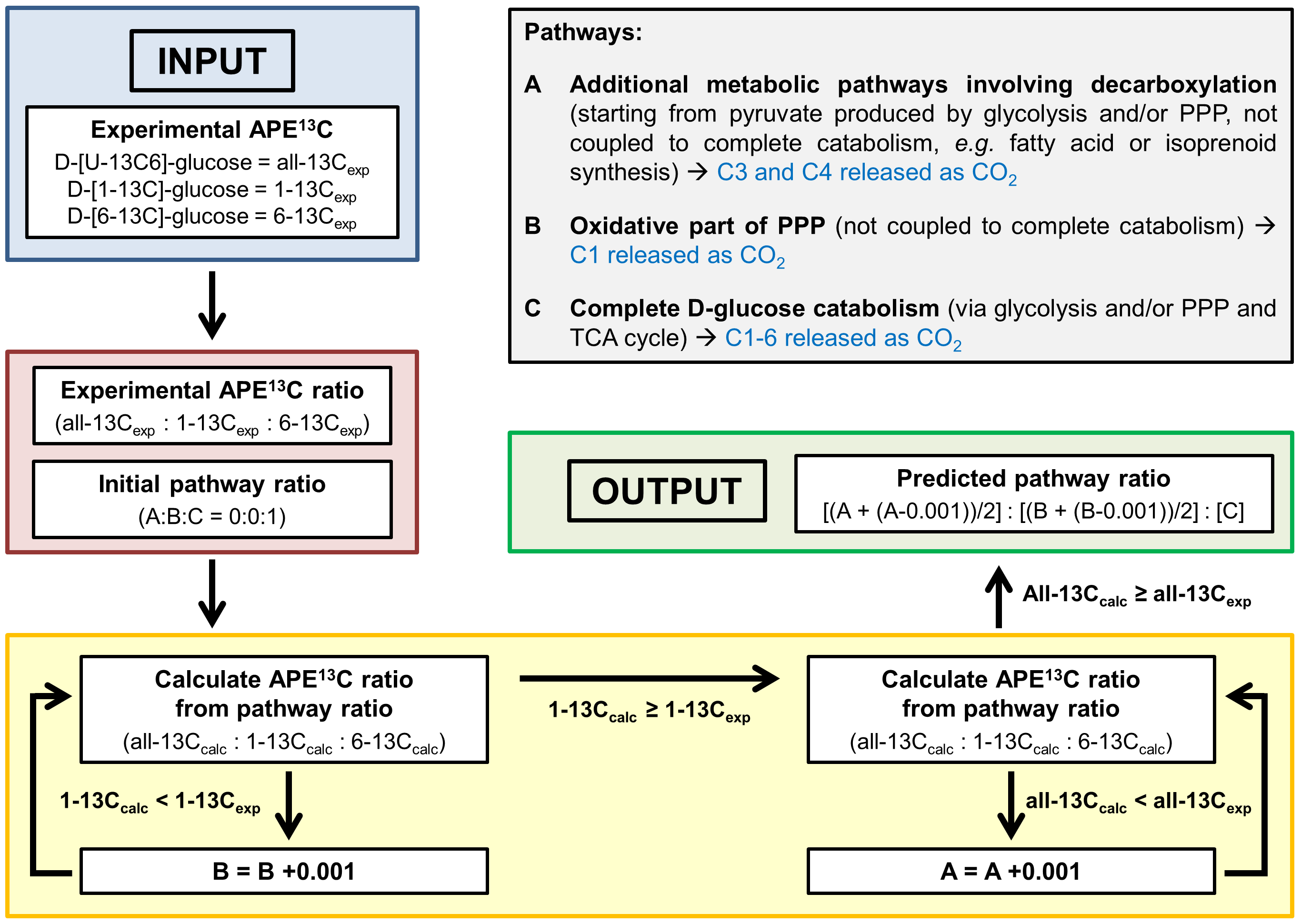

Supplement: Figure S4 — Working scheme for the estimation of the contributions of different metabolic pathways to 13CO2 release. In respect to the release of certain carbon atoms from D-glucose as CO2, three major metabolic scenarios (A–C) were distinguished, which are indicated in the gray box and will here be termed “pathways” for simplicity. The pathway ratio was calculated based on the APE13C in CO2 observed for incubations in media with different labeled D-glucose isotopologs (blue box). Initially, these experimental values were used to calculate an experimental APE13C ratio and the pathway ratio was set to 0∶0∶1 (A∶B∶C), assuming that all D-glucose is completely catabolized (red box). Based on this pathway ratio a corresponding expected ratio of APE13C for the differently labeled substrates was calculated, assuming that pathways A, B, and C lead to a release of 1.83, 1, and 6, respectively, carbons from D-[U-13C6]-glucose, 0, 1, and 1, respectively, carbons from D-[1-13C]-glucose, and 0, 0, and 1, respectively, carbons from D-[6-13C]-glucose. The assumed release of 1.83 carbon atoms per molecule D-[U-13C6]-glucose by pathway A was based on the assumption that pyruvate, as starting molecule for scenario A, is produced with equal probability from glycolysis and PPP activity, the latter of which already releases carbon 1 as CO2 and thus yields a lower amount of pyruvate per molecule glucose. The calculated expected APE13C ratio was then compared to the experimental APE13C ratio. This was followed by stepwise adjustments of B and A until the expected APE13C ratio coincided with the experimentally observed ratio (yellow box). The finally obtained pathway ratio represents an estimation of the contributions of the three considered metabolic scenarios to 13CO2 release by host-free P. amoebophila EBs (green box). (TIF) [file ppat.1003553.s004.tif]

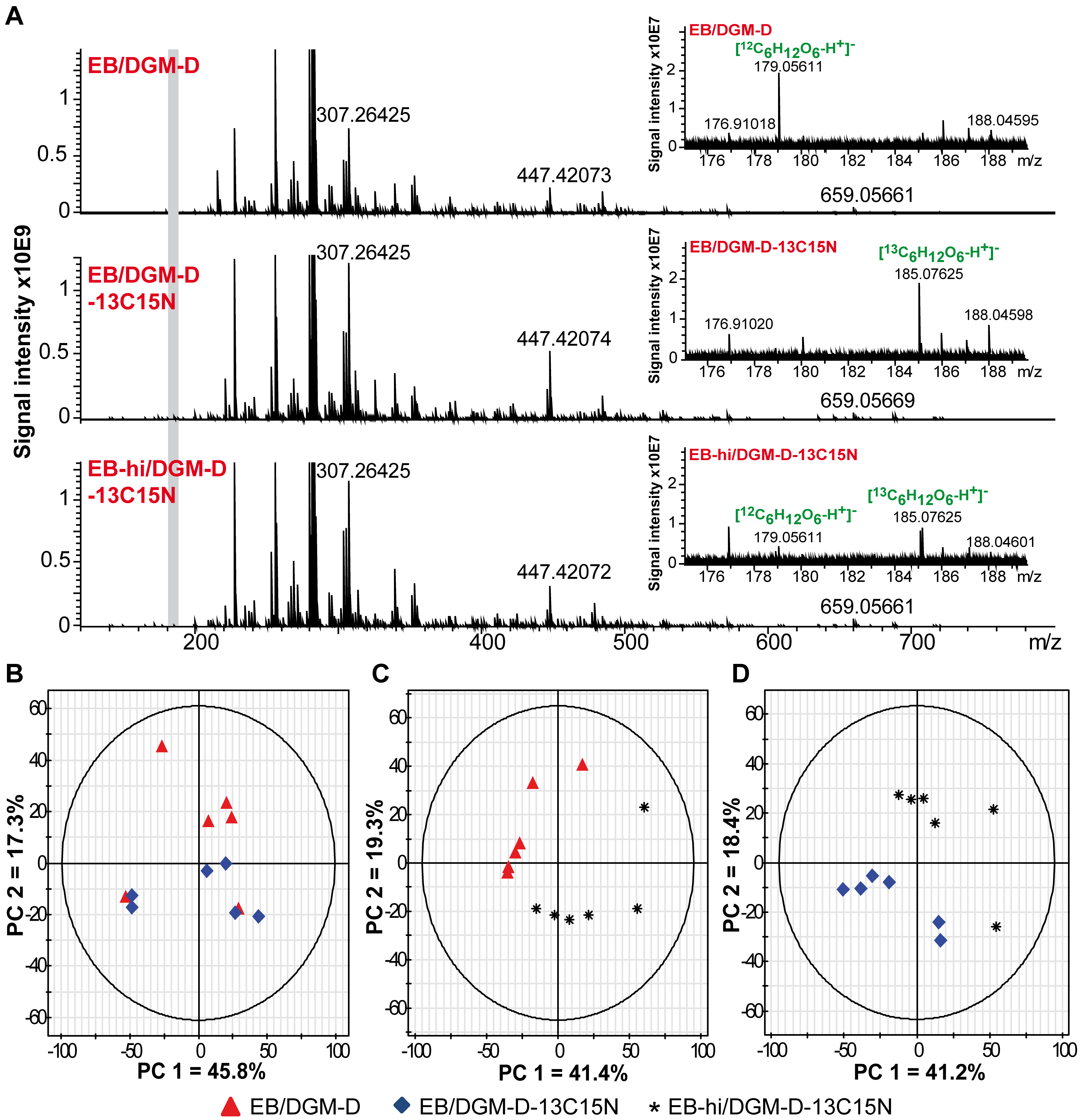

Supplement: Figure S5 — Comparison of ICR/FT-MS metabolite profiles from DGM-D- and DGM-D-13C15N-incubated living and heat-inactivated EBs. In (A) representative ESI(-)ICR/FT-MS spectra of DGM-D- and DGM-D-13C15N-incubated living bacteria, as well as of DGM-D-13C15N-incubated inactivated bacteria, are shown. Spectra illustrate the m/z range of 150–800. In addition, an enlarged view on the m/z range of 175–190 (indicated in gray in the overview spectra), is displayed. A comparison of spectra from living bacteria incubated in the two different media revealed a shift of the D-glucose peak from m/z ∼179 (12C-D-glucose) to m/z ∼185 (13C-D-glucose), while the remaining pattern was unaltered. The exchange of D-glucose and L-phenylalanine in the incubation medium against their stable isotope-labeled analogs thus did not profoundly change the metabolite profile of P. amoebophila EBs. This visual impression, as well as the effect of heat-inactivation, was further tested by pairwise PCA comparisons of spectra from DGM-D- and DGM-D-13C15N-incubated living bacteria (B), and of spectra from DGM-D-1315N-incubated inactivated bacteria with those from DGM-D-incubated living EBs (C) or DGM-D-13C15N-incubated living EBs (D). Note that this analysis revealed a separation of spectra from living and dead bacteria, whereas no separation could be observed for samples from living bacteria incubated in the two different media. (TIF) [file ppat.1003553.s005.tif]

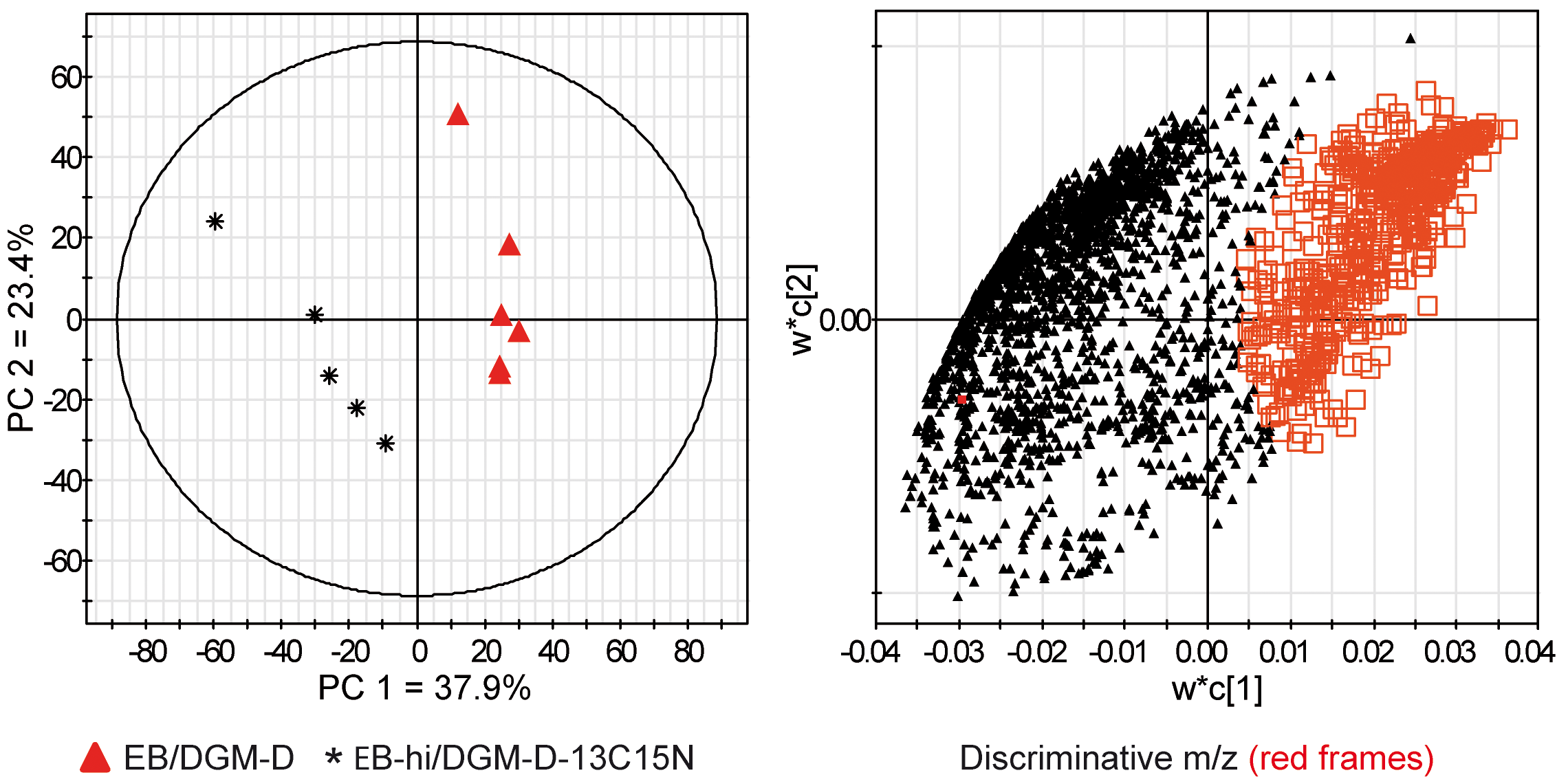

Supplement: Figure S6 — PLS-DA analysis for the extraction of metabolites discriminative for living compared to heat-inactivated EBs. ICR/FT-MS spectra were analyzed by PLS-DA to extract the most discriminative compounds characterizing living compared to inactivated EBs. The PLS-DA model included data from DGM-D-incubated living and DGM-D-13C15N-incubated inactivated EBs. The assignment of discriminative and non-discriminative metabolites to KEGG pathways is displayed in Fig. 4. (TIF) [file ppat.1003553.s006.tif]

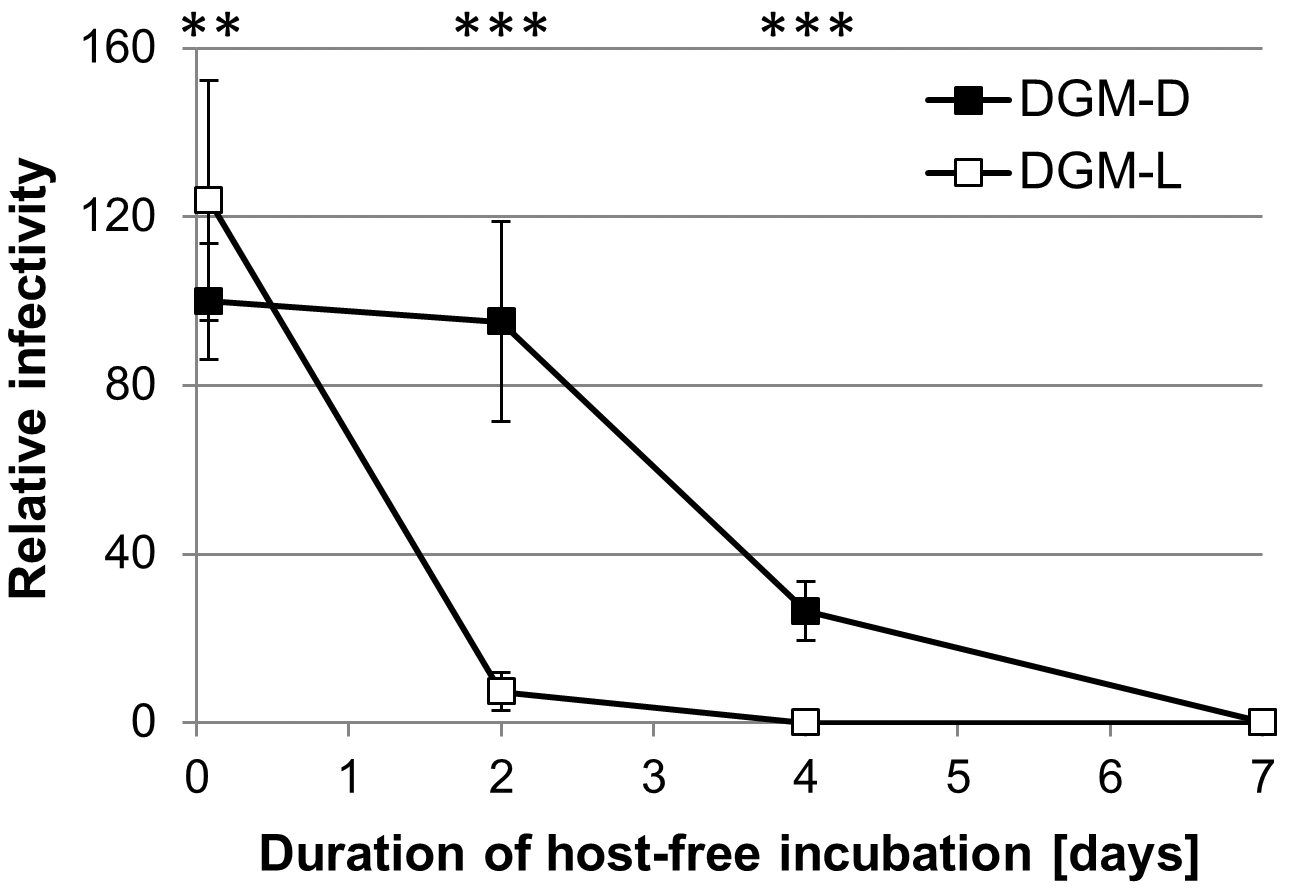

Supplement: Figure S7 — Lack of infectivity restoration by addition of D-glucose to starved P. amoebophila . P. amoebophila were harvested from amoebal host cell cultures followed by host-free incubation for the indicated periods of time in DGM-D or DGM-L. Subsequently, bacteria incubated in DGM-L were supplemented with D-glucose (83.2 mM final concentration). After an additional incubation of the bacteria for 15 min (27°C, 200 rpm), amoebae were infected at a MOI of 4.3 and the percentage of infected cells was assessed at 48 h p.i. after detection of intracellular bacteria by FISH. The observed infectivity, relative to that observed for 2 h incubation in DGM-D, is shown. Data represent means and standard deviations of three independent experiments, each consisting of three replicate host-free incubations. For each sample at least 600 cells were counted. Statistically significant differences in infectivity observed between the incubation media at selected time points after start of host-free incubation are indicated (t-test; ***, p≤0.001; **, p≤0.01). (TIF) [file ppat.1003553.s007.tif]
