# Supplementary material for: Metabolic Features of Protochlamydia amoebophila Elementary Bodies – A Link between Activity and Infectivity in Chlamydiae
Source: PLoS Pathog. 2013 Aug 8;9(8):e1003553. doi: 10.1371/journal.ppat.1003553 (PMC3738481; doi:10.1371/journal.ppat.1003553)
Supplement: Table S1 — Non-annotated 13C-labeled metabolites detected by ICR/FT-MS in DGM-D-13C15N-incubated EBs. (DOCX) [file ppat.1003553.s008.docx]

**Table S1: Non-annotated ^13^C-labeled metabolites detected by ICR/FT-MS in DGM-D-13C15N-incubated EBs.**

| **Detected m/z (DGM-D)** | **Detected m/z (DGM-D-13C15N)** | **Number of ^13^C atoms** | **Calculated elemental composition (error < 0.1 ppm)** |
| --- | --- | --- | --- |
| 178.9726907 | 180.9794218 | 2 | C_9_H_4_ClS |
| 190.9727033 | 193.98278 | 3 | C_10_H_4_ClS |
| 217.0370256 | 223.0571771 | 6 | - |
| 234.9836156 | 238.9970475 | 4 | C_7_H_8_O_5_PS; C_8_H_8_ClO_4_S |
| 236.9313314 | 238.9380444 | 2 | - |
| 269.0878227 | 275.1079733 | 6 | C_9_H_17_O_9_ |
| 272.9914136 | 279.0115267 | 6 | C_11_H_11_ClO_2_PS |
| 274.1230861 | 276.1298 | 2 | C_11_H_20_N_3_O_3_S |
| 282.0384406 | 285.0485507 | 3 | C_8_H_13_NO_8_P; C_9_H_13_ClNO_7_ |
| 290.1180109 | 293.1281 | 3 | C_11_H_2_0N_3_O_4_S; C_10_H_17_N_4_O_6_; C_12_H_15_N_7_P |
| 299.0150179 | 305.03512 | 6 | C_13_H_12_ClO_4_S; C_12_H_13_O_5_PS |
| 302.5335167 | 305.5435667 | 3 | - |
| 304.1336544 | 307.1437067 | 3 | C_12_H_22_N_3_O_4_S; C_15_H_19_ClN_5_ |
| 318.1129306 | 321.1229983 | 3 | C_15_H_17_N_5_O |
| 320.1285839 | 323.1385917 | 3 | C_7_H_19_N_11_PS; C_12_H_22_N_3_O_5_S; C_15_H_19_ClN_5_O |
| 328.94209 | 331.952094 | 3 | - |
| 348.0766194 | 350.083372 | 2 | - |
| 350.9241017 | 353.9341257 | 3 | - |
| 408.1446006 | 414.1647475 | 6 | C_18_H_23_ClN_5_O_4_ |
| 421.4051174 | 423.4117864 | 2 | C_28_H_53_O_2_ |
| 429.314003 | 438.3441471 | 9 | - |
| 435.2963511 | 441.3164287 | 6 | C_22_H_43_O_8_ |
| 435.334106 | 438.3441471 | 3 | C_24_H_43_N_4_O_3_ |
| 459.2963586 | 465.316444 | 6 | C_24_H_43_O_8_ |
| 461.3119945 | 468.33544 | 7 | C_24_H_45_O_9_ |
| 461.3119945 | 467.3320894 | 6 | C_24_H_45_O_8_ |
| 463.3276456 | 469.3476929 | 6 | C_24_H_47_O_8_ |
