# Supplementary material for: Metabolic Features of Protochlamydia amoebophila Elementary Bodies – A Link between Activity and Infectivity in Chlamydiae
Source: PLoS Pathog. 2013 Aug 8;9(8):e1003553. doi: 10.1371/journal.ppat.1003553 (PMC3738481; doi:10.1371/journal.ppat.1003553)
Supplement: Table S2 — Media for extracellular incubation of P. amoebophila . (DOCX) [file ppat.1003553.s009.docx]

**Table S2: Media for extracellular incubation of *P. amoebophila*.**

|  | **Glucose** | |  | **Phenylalanine** | |
| --- | --- | --- | --- | --- | --- |
| **Medium** | **Isotopolog/ Stereoisomer** | **Concentration [mM]** |  | **Isotopolog/ Stereoisomer** | **Concentration [mM]** |
| DGM-D | D-glucose | 83.2 |  | L-phenylalanine | 5.4 |
| DGM-D/2 | D-glucose | 41.6 |  | L-phenylalanine | 5.4 |
| DGM-L | L-glucose | 83.2 |  | L-phenylalanine | 5.4 |
| DGM-DL | D-glucose, L-glucose | 83.2 (both) |  | L-phenylalanine | 5.4 |
| DGM-D6P | D-glucose-6-phosphate | 83.2 |  | L-phenylalanine | 5.4 |
| DGM-DD6P | D-glucose, D-glucose-6-phosphate | 83.2 (both) |  | L-phenylalanine | 5.4 |
| DGM-D-13C | D-[U-13C6]-glucose (99%) | 83.2 |  | L-phenylalanine | 5.4 |
| DGM-D-1-13C | D-[1-13C]-glucose (98-99%) | 83.2 |  | L-phenylalanine | 5.4 |
| DGM-D-6-13C | D-[6-13C]-glucose (99%) | 83.2 |  | L-phenylalanine | 5.4 |
| DGM-D-13C15N | D-[U-13C6]-glucose (99%) | 83.2 |  | L-[U-13C9,15N]-phenylalanine (97-99%) | 5.4 |

All listed media are based on the chemically defined *Acanthamoeba* medium DGM-21A [[1](#_ENREF_1)], but were additionally supplemented with 0.25 g/l NaHCO_3_. Included isotopologs/stereoisomers of glucose and phenylalanine, as well as their concentration, are indicated.

**References**

1. Schuster FL (2002) Cultivation of pathogenic and opportunistic free-living amebas. Clin Microbiol Rev 15: 342-354.
